# Supplementary material for: Spatial heterogeneity of the nonlinear impact of built environment on suburban railway commuting willingness: Taking Guiyang Loop Line Railway as an example
Source: PLoS One. 2026 Apr 7;21(4):e0346358. doi: 10.1371/journal.pone.0346358 (PMC13056205; doi:10.1371/journal.pone.0346358)
Supplement: Attachment — Submitted filename: Review PONE-D-25-62306.pdf [file pone.0346358.s001.pdf]

PONE-D-25-62306 Spatial heterogeneity of the nonlinear impact of built environment on suburban railway commuting willingness

This study takes the Guiyang Loop Line Railway as a case study and applies XGBoost and ICE models to examine the non-linear effects and spatial heterogeneity of the built environment on commuters' willingness to use the Loop Line Railway. The research topic is innovative, and the integration of multiple data sources adds interest to the study. However, despite presenting a relatively complete analytical framework, the manuscript still exhibits several notable shortcomings that should be addressed.

General comment

1. It is recommended that line numbers be added to the manuscript upon resubmission to make the review process more convenient and help provide more precise comments.

Abstract

2. The findings section of the abstract is overly generic. Although the study identifies several meaningful and distinctive patterns, the abstract merely states the existence of non-linear impacts and threshold effects. More specific and concrete findings should be highlighted to better reflect the study's contribution.

Introduction

3. The authors provide a detailed description of the metro system in Guiyang, which helps readers understand the background of the study area. However, the discussion of the key issues, such as "persistent issues of low utilization and sparse passenger numbers" and "core functions remain underutilized", is insufficient. Providing empirical evidence (e.g., actual ridership statistics, service frequency data) or concrete examples would better justify the selection of the Guiyang Loop Line Railway as the study case.

4. On pages 4–5, several factors contributing to the low utilization of the Loop Line Railway are listed (e.g., infrequent services, inadequate infrastructure, cumbersome transfers, and costs). However, built environment factors are barely discussed. Given that the core focus of the study is effects of the built environment on commuter willingness, the authors should clearly explain why built environment characteristics are particularly relevant in explaining low ridership. Strengthening this conceptual linkage would enhance the necessity of the study.

5. Although commuter willingness is the central dependent variable, the literature review does not sufficiently cover previous studies on commuter willingness, including relevant theoretical frameworks and methodological approaches.

Data Sources

6. The description of the survey data is insufficiently detailed. Key information is missing, including the survey period, the format of the questionnaire, the measurement scale used for the items (e.g., five-point Likert scale), and whether the survey was conducted in paper-based or electronic form. If an online survey was used, the specific platform should also be stated.

7. A total of 700 questionnaires were returned, but only 451 were used for model estimation, resulting in a valid response rate of 64.43%. The data cleaning process and exclusion criteria should be clearly described to ensure data processing is conducted rigorously.

8. Table 1 presents the measurement items for commuter willingness. However, it is unclear how these items were selected and on what basis. Additional explanations or supporting references should be provided.

ICE Model

9. On page 14, please check the interpretation of the m-value in Equation (7). Specifically, it should be clarified whether a higher m-value indicates a greater degree of non-linearity in the effect or a greater overall magnitude of influence?

Spatial Characteristics of Threshold Effect

10. It is recommended that the authors include comparisons with findings from previous studies to better highlight the novel contributions of the current research.

11. The first sentence on pages 14-15 is incomplete and should be revised for clarity.

12. On page 17 The sentences beginning with "*When built environment variables exhibit threshold effects…*" is strikingly similar in wording to a Chinese-version publication. The authors should either explicitly cite the original source or rewrite the paragraph to avoid concerns regarding textual similarity.

13. The statement on page 18, "*This suggests that in peripheral urban areas, changes in population density have a relatively limited impact on commuting willingness, with a less pronounced threshold effect*", is not sufficiently precise. A weaker threshold effect does not necessarily imply a limited overall impact. Similar issues of imprecise appear elsewhere in the manuscript, requiring more careful wording to ensure conceptual and empirical accuracy.

Spatial Analysis of Threshold Effects

14. The section title "*Spatial Analysis of Threshold Effects*" does not accurately reflect the content of the section, which primarily discusses non-linear impacts rather than threshold effects.

15. The manuscript lacks a discussion of limitations and future research directions, which should be added.

Other Issues

16. The manuscript structure is incomplete and does not fully conform to the conventions of academic writing in international journals. For example, the paper lacks: a standalone literature review section that clearly identifies research gaps, a detailed description of the questionnaire design and data collection procedures, and a section discussing limitations and directions for future research.

17. Although the language is generally concise, the manuscript contains multiple informal expressions and instances of Chinglish. In addition, many sentences are low in information density and overly

generic (e.g., "*The questionnaire content is as shown in Table 1*" or repeated vague expressions like "*However, indiscriminately increasing… may/could…*"). The language should be carefully revised to improve precision.
