# Supplementary material for: Spatial heterogeneity of the nonlinear impact of built environment on suburban railway commuting willingness: Taking Guiyang Loop Line Railway as an example
Source: PLoS One. 2026 Apr 7;21(4):e0346358. doi: 10.1371/journal.pone.0346358 (PMC13056205; doi:10.1371/journal.pone.0346358)
Supplement: Attachment — Submitted filename: Response to Reviewers.docx [file pone.0346358.s003.docx]

**Point-to-Point Responses to Reviewers and Editor**

The authors are very thankful to the anonymous referees for the detail analysis and comments on the work. A number of interesting issues, questions and useful advises have been raised through the comments. In the past days, we have considered very carefully and seriously the questions and suggestions enclosed in the review and made modifications, adjustments, elaborations, etc. Accordingly, to make this paper more convincing, explicit, complete and acceptable. In the following the authors respond to each of their concerns and advises in an itemized fashion.

***Responses to comments from Reviewer #* 1**

## **Reviewers comment 1**

**In the introduction, the authors stated that residents in Guiyang face limited transport options and poor road networks. Statistics and references are needed to support such claims.**

## **Response to comment 1**

We appreciate the reviewer’s constructive suggestions regarding the description of Guiyang’s current transportation status in the Introduction. To strengthen the empirical evidence and persuasiveness of our argument, we have incorporated reports from authoritative media outlets concerning Guiyang’s challenging road network conditions in the revised manuscript. The specific details are provided as follows (**see Lines 114–124 in manuscript**):

It is indicated in the Guiyang “14th Five-Year” Special Transportation Construction Plan that the road network structure in the central urban area remains incomplete, characterized by a low proportion of secondary and branch roads, poor network circulation, and a severe shortage of overall road network density. Consequently, further improvement is required for the traffic carrying capacity [15]. By October 2024, an increase of approximately 20,000 motor vehicles per month was recorded in Guiyang, while the urban road network density stood at only 6.7 km/km², far below the national standard of 8 km/km². This imbalance between vehicle growth and road supply has led to a pronounced disparity between vehicle proliferation and road infrastructure availability, further constraining the efficiency of cross-regional commuting[16],

## **Reviewers comment 2**

**What is Loop Line railway, and how it differs from other railways? This requires further explanation.**

## **Response to comment 2**

We sincerely thank the reviewer for this insightful comment. We agree that the initial conceptual definition of the 'Loop Line Railway' and its distinction from other urban rail transit modes were not sufficiently articulated. In response, we have expanded the Introduction to include a more rigorous definition, functional positioning, and a comparative analysis of the Loop Line Railway versus other railway types. These additions aim to enhance conceptual clarity and the overall readability of the manuscript. The revised text is as follows (**see Lines 58–73 in manuscript**)：

Suburban railways, often termed commuter rail or suburban rail, are categorized as a form of passenger rail system positioned between urban rail transit (e.g., metro systems) and intercity railways; they are characterized by commuter-oriented services, relatively high operating speeds, and large transport capacity. As a long-distance commuting mode, a crucial role is played by suburban railways in connecting central urban areas with peripheral suburbs and in supporting the development of a “one-hour commuting circle” [1]. In comparison to conventional urban rail transit, a wider spatial area is typically served by suburban railways, which feature larger station spacing, higher operating speeds, and lower service frequencies. They are primarily designed to accommodate medium-to long-distance commuting between central cities and surrounding suburban areas or satellite towns. Their functional orientation is focused on establishing efficient commuting corridors within metropolitan areas[2-3]. In this context, a significant contribution is made by suburban railways toward relieving functional pressure in central urban areas, shaping rational commuting structures, facilitating convenient daily mobility, and improving accessibility in peripheral urban regions [4].

## **Reviewers comment 3**

**The underutilisation of the Loop Line railway is a very specific case. How it relates to the broader context and literature? The authors need to better position the current study in the literature.**

## **Response to comment 3**

We sincerely thank the reviewer for this critical insight. We agree that the underutilization of the Guiyang Loop Line Railway should not be viewed as an isolated case but rather needs to be discussed within the broader context of regional and suburban rail research. Following this suggestion, we have systematically expanded the literature review in the Introduction to emphasize the universal significance of this study. Specifically, the revised manuscript now highlights that the underperformance of commuting functions in regional railways is a common challenge faced by multiple cities in China, rather than an issue unique to Guiyang. For example(**see Lines 79–97, 145-148 in manuscript**):

Within the Chinese context, intended commuting functions have not been fully realized by many operational suburban railway lines, which commonly face problems of low ridership and underutilization [7-8]. For example, tourist travel has been primarily served by Beijing’s S2 Line since its opening, with commuting trips accounting for only a minor share of total ridership[9].

Concurrently, we reviewed the mainstream explanatory pathways in existing research regarding this issue and, on this basis, further identified the limitations of current studies：

To explain these phenomena, the limited commuting performance of suburban railways has been mainly attributed by previous studies to operational or institutional factors, including inappropriate station location, relatively high fares, long waiting times, and insufficient supporting infrastructure [10-12]. However, for suburban railways characterized by larger station spacing and broader service catchments, the conversion of commuting willingness into actual ridership is not only dependent on train operating services but is also strongly constrained by the built environment conditions surrounding residents’ places of dwelling. In contrast to urban metro systems, a higher sensitivity to first- and last-mile travel conditions is exhibited by commuting behavior on suburban railways. When feeder bus services are insufficient, road network connectivity is poor, or station accessibility is low, the adoption of suburban railways as a regular commuting mode may be found difficult by residents, even when latent commuting willingness exists. Consequently, persistently low utilization of such lines is often observed.

So the Guiyang Loop Line Railway—a representative example of a loop-type suburban rail system—is presented as a valuable empirical context for examining how built environment factors influence commuters’ willingness to use suburban railways.

Through these additions, we have situated the research on the Guiyang Loop Line Railway within a broader regional rail commuting framework, highlighting its theoretical value in extending the explanatory perspectives of existing literature.

## **Reviewers comment 4**

**The authors mentioned previous research on railway usage quite late into the introduction. The structure here can be considerably improved by reorganizing the logic, from broader picture to the specific case of Guiyang. It is also suggested to justify the case study as a valuable addition to the literature.**

## **Response to comment 4**

We appreciate the reviewer’s constructive comments. We agree that there was room to optimize the placement of the literature review and the overall logical flow within the Introduction. Following your suggestion, the revised manuscript has undergone a systematic restructuring of the Introduction, reorganizing the content according to the logical sequence of "Research Background—Existing Research—Research Gaps—Case Introduction—Research Objectives" (**see Lines 74–87,101-152 in manuscript**).

In the revised version, we begin from a broader research background and systematically review the role of regional railways in supporting long-distance commuting systems within metropolitan areas, as well as the challenges currently faced in their operation:

With the continuous expansion of urban space and the deepening spatial mismatch between residential and employment locations, increasing scholarly attention has been attracted by suburban railways worldwide. The potential role of suburban rail systems in supporting metropolitan commuting structures has been highlighted by existing studies, viewed from perspectives such as urban land-use optimization, spatial structure refinement, and regional integration[5-6]. However, within the Chinese context, intended commuting functions have not been fully realized by many operational suburban railway lines, which commonly face problems of low ridership and underutilization [7-8]. For example, tourist travel has been primarily served by Beijing’s S2 Line since its opening, with commuting trips accounting for only a minor share of total ridership[9].

Subsequently, we further summarized the primary limitations of current research:

To explain these phenomena, the limited commuting performance of suburban railways has been mainly attributed by previous studies to operational or institutional factors, including inappropriate station location, relatively high fares, long waiting times, and insufficient supporting infrastructure [10-12].

However, for suburban railways characterized by larger station spacing and broader service radii, focusing solely on the supply of operational services often fails to fully reveal the underlying mechanisms behind limited commuting functionality and sparse passenger flow. Although some scholars have noted the influence of the built environment on residents' commuting behavior, the attention given to built environment factors remains significantly insufficient:

On the one hand, a station-area (destination-end) perspective has been primarily adopted in prior research, emphasizing land use patterns or development intensity around stations; conversely, systematic empirical evidence regarding the effect of residential-end built environment characteristics on commuters’ mode choice remains scarce[13]. On the other hand, most existing studies are based on large cities with relatively simple topographic conditions[14]. Mountainous cities, where urban spatial structure is strongly constrained by natural terrain, have received far less attention, and the applicability of existing findings to such contexts has yet to be adequately examined.

On this basis, we introduce the Guiyang Loop Line Railway as the research case:

Guiyang, situated in southwest China, is exemplified as a typical mountainous city characterized by steep slopes and complex terrain. As of 2025, only Metro Lines 1, 2, 3, and the S1 Line had been commissioned in Guiyang, resulting in limited coverage of the urban rail transit network. Consequently, heavy reliance is placed by residents on other public transport modes for cross-regional commuting. Simultaneously, traffic congestion has been exacerbated by factors such as insufficient road network connectivity. It is indicated in the Guiyang “14th Five-Year” Special Transportation Construction Plan that the road network structure in the central urban area remains incomplete, characterized by a low proportion of secondary and branch roads, poor network circulation, and a severe shortage of overall road network density. Consequently, further improvement is required for the traffic carrying capacity [15]. By October 2024, an increase of approximately 20,000 motor vehicles per month was recorded in Guiyang, while the urban road network density stood at only 6.7 km/km², far below the national standard of 8 km/km². This imbalance between vehicle growth and road supply has led to a pronounced disparity between vehicle proliferation and road infrastructure availability, further constraining the efficiency of cross-regional commuting[16],

To address these challenges, China’s first urban loop suburban railway—the Guiyang Loop Line Railway—was officially inaugurated in Guiyang on March 30, 2022. The line comprises a total length of approximately 113 km and includes 17 stations. Positioned as an important commuting corridor connecting multiple residential areas with the central urban area, the Loop Line Railway was designed to promote the development of the urban transport system, enhance connectivity between the city center and peripheral clusters, and support the construction of a “Guiyang–Gui’an one-hour commuting circle”. However, in comparison with its planned positioning and functional expectations, problems of low utilization and insufficient ridership have been faced by the Loop Line Railway in actual operation.

Against this background, the Guiyang Loop Line Railway—a representative example of a loop-type suburban rail system—is presented as a valuable empirical context for examining how built environment factors influence commuters’ willingness to use suburban railways. A detailed investigation into how variations in residential-side built environment characteristics shape first- and last-mile travel conditions—consequently affecting residents’ willingness to commute via the Loop Line Railway—is essential for improving operational efficiency and addressing the limitations of existing studies in terms of spatial perspective and topographic context.

Through the aforementioned adjustments, we have further strengthened the positioning of this study within the existing literature framework and emphasized its theoretical and practical significance.

## **Reviewers comment 5**

**How are the sample and survey stations determined? This requires explanation and justification. This should bring forward in the methodological section.**

## **Response to comment 5**

We appreciate the reviewer’s comments. We agree that the explanation regarding the selection of samples and survey stations in the original manuscript was not sufficiently centralized, and the methodological justification required strengthening. Following your suggestion, a new subsection titled " Sample Selection and Survey Site Determination" has been added to the Research Design section of the revised manuscript. This subsection provides supplementary details on the principles, screening process, and rationale for determining the samples and survey stations. The specific additions are as follows (**see Lines 259–308 in manuscript**):

Sample Selection and Survey Site Determination

This study investigates residents’ willingness to commute via the Guiyang Loop Line Railway. Given that suburban railways primarily facilitate medium-to long-distance commuting between urban cores and peripheral areas, they are characterized by large inter-station spacing and significant reliance on access and egress connections. Consequently, conventional catchment area delineations based on walking distances, which are commonly applied to urban metro systems, are not directly applicable in this context. Previous studies indicate that users of suburban or commuter rail systems rely heavily on cycling, feeder buses, and other modes—in addition to walking—to complete first- and last-mile trips; as a result, acceptable access distances substantially exceed the typical walking thresholds observed for metro systems[47]. Further empirical evidence suggests that commuters’ tolerance regarding access and egress is constrained more significantly by temporal thresholds than by physical distance alone [48]. Field investigations conducted for this study indicate that, in commuting contexts, passengers generally accept access and egress travel times of 15–20 minutes, which corresponds approximately to a spatial radius of 2–3 km. In comparison to larger catchment definitions, a 3 km buffer effectively captures the primary residential clusters surrounding stations while excluding distant samples that exhibit weak relevance to suburban rail commuting decisions. Therefore, considering the functional characteristics of suburban railways, station spacing, and commuters’ access behavior, the research area in this study is defined as a 3 km radius around each station.

The survey encompassed all operational stations along the Guiyang Loop Line Railway.To ensure spatial representativeness, questionnaire allocation was proportional to the population size within each station’s 3 km buffer: stations characterized by higher population density and greater functional centrality were assigned larger sample sizes, whereas peripheral suburban stations were allocated fewer questionnaires. The specific survey locations and study areas are illustrated in Figure 1.

Fig. 1 Schematic diagram of survey points and study area

The questionnaire design was centered on the mechanisms underlying residents’ willingness to commute via the Loop Line Railway. Data regarding respondents’ commuting characteristics, alongside subjective perceptions of service performance, station conditions, and associated railway attributes, were collected. Prior to the full-scale survey, a pilot study was conducted to refine and simplify the wording, thereby minimizing ambiguity and enhancing response quality. The primary survey was administered between April and May 2024 via face-to-face paper-based questionnaires distributed within a 3 km radius of each station. In total, 750 questionnaires were distributed. Following the exclusion of questionnaires with incomplete responses or significant logical inconsistencies, 700 valid questionnaires were retained. During the model estimation phase, a secondary screening of the sample was performed to ensure the analysis adequately captured the differential effects of the built environment on commuting willingness across varied spatial contexts. Specifically, a subset of 451 questionnaires was selected for analysis, drawn from six representative stations: Guiyang Station, Guiyang North Station, Baiyun North Station, Jinyang South Station, Gui’an Station, and Huaxi South Station. Substantial variations in built environment characteristics, spatial location, infrastructure provision, functional roles, and ridership levels are exhibited by these stations. Collectively, they represent core transportation hubs, regional sub-centers, and standard functional stations, reflecting a spatial gradient spanning from the urban center to suburban and peripheral zones. This sampling strategy facilitates a comprehensive examination of the heterogeneous effects of the built environment on individuals’ willingness to commute via the Loop Line Railway across diverse urban contexts.

## **Reviewers comment 6**

**It is unclear how the subjective statements are selected. It seems that some behavioural and social psychological theories might be considered. But a discussion of the relevant theories and the underlying framework are completely missing.**

## **Response to comment 6**

We appreciate the reviewer’s significant comment. We agree that the explanation regarding the sources of subjective perception variables and their theoretical basis was insufficient in the original manuscript, which could lead to ambiguity concerning the selection criteria for questionnaire items. In the revised manuscript, we have systematically elaborated on the relevant theories within the Literature Review section (**see Lines 162–183 in manuscript**). Concurrently, a new section titled "Theoretical Foundations and Questionnaire Design" has been added **(**(**see Lines 309–343 in manuscript**) to provide a systematic supplement on the theoretical origins and construction logic of the subjective statement items. As this suggestion aligns with the Editor’s Comment No. 8, we have adopted the same response.。

Theoretical Foundations and Questionnaire Design

Individual commuting mode choice is influenced not only by objective built environment conditions but is also strongly contingent upon individuals’ subjective perceptions of travel convenience, accessibility, and service quality. The Theory of Planned Behavior (TPB) [18] and the Technology Acceptance Model (TAM)[24] are recognized as widely applied frameworks for explaining travel mode choice behavior. Consequently, these two theories are adopted in this study as the theoretical foundation for analyzing residents’ commuting behavior. In TPB, it is emphasized that individuals’ behavioral intention is jointly determined by attitudes, subjective norms, and perceived behavioral control[49]. The focus of TAM lies in individuals’ subjective evaluations of the perceived usefulness and perceived ease of use of transport systems. This model is considered particularly suitable for explaining acceptance behavior toward emerging transport modes[50]. Building on these frameworks—and given the high dependence of suburban railway commuting on station-area environments and service experiences[51] —satisfaction with the Loop Line Railway, perceived station conditions, and perceived road conditions are introduced as contextual extension variables. These variables are incorporated to address the limitations of traditional behavioral theories in capturing spatial and infrastructure - related factors. Variable definitions and measurements are summarized in Table 1.

The questionnaire comprises nine dimensions and 32 measurement items. Perceived usefulness measures residents’ evaluations of the Loop Line Railway in terms of commuting efficiency, economic benefits, and improvements in physical and mental well-being. Perceived ease of use reflects residents’ perceptions of convenience in accessing stations, obtaining operational information, and purchasing tickets. Subjective norms capture the influence of online publicity, fare policies, and attitudes of family members and friends on commuting choices. Satisfaction with the Loop Line Railway evaluates timetable arrangements for commuting, riding comfort, and service quality. Perceptions of station conditions and road conditions reflect the effects of station layout, feeder facilities, and access road characteristics on commuting willingness. Perceived behavioral control assesses residents’ judgments regarding their ability to use the Loop Line Railway and tolerate relatively complex commuting procedures. Attitude represents residents’ overall evaluations of the convenience, usefulness, and comfort of commuting by the Loop Line Railway. Finally, commuting willingness reflects residents’ intentions to choose the Loop Line Railway for commuting under current conditions and to recommend it to others.Table 1 Commuter willingness survey questionnaire for the Loop Line Railway

Table 1 Commuter willingness survey questionnaire for the Loop Line Railway

| Measurement dimension | Measurement Item |
| --- | --- |
| Perceived usefulness | Commuting efficiency. |
|  | Economic benefits |
|  | Well-being improvement |
| Perceived Usability | Access convenience |
|  | Learning ease |
|  | Information accessibility |
|  | Ticketing convenience |
| Subjective Norms | Online publicity impact |
|  | Fare elasticity |
|  | Peer usage |
|  | Social imitation |
|  | Social encouragement |
| Satisfaction with the Loop Line Railway | Schedule suitability |
|  | Comfortable travel environment. |
|  | Reasonable commuter fares. |
|  | Service attitude |
|  | Operation frequency |
| Station-aware | Station layout |
|  | Feeder facilities |
|  | Land-use development |
| Road Condition Perception | Road gradient |
|  | Road circuity |
|  | Road width |
| Perceptual-behavioural control | Behavioral ease |
|  | App proficiency |
|  | Procedural resilience |
| Attitude | Commuting convenience |
|  | Practicality |
|  | Perceived comfort |
| Commuting willingness | Operational satisfaction |
|  | Modal preference |
|  | Recommendation intention |

## **Reviewers comment 7**

**It is unclear how the proximity to bus stops may influence the usage of metro. The bus may serve as a feeder line to the railway. But it is unknown whether it is the case, and what is the proportion of intermodal transit users in Guiyang. The argued link between proximity to bus stops and walkability is weak by comparison.**

## **Response to comment 7**

We appreciate the reviewer’s insightful inquiry regarding the underlying mechanism of the "Distance to Nearest Bus Stop" variable. We agree that, to strictly identify whether buses serve as a feeder mode for the regional railway, it would ideally be necessary to possess specific transfer proportions or trip-chain data for residents' multi-modal commutes. However, due to survey constraints, this study was unable to obtain precise information regarding the proportions of different transportation mode combinations, which represents an objective limitation of this research. Consequently, we have cautiously refined the interpretation of the "Distance to Nearest Bus Stop" variable in the revised manuscript. We do not interpret this variable as a definitive feeder relationship between the bus system and the Loop Line Railway, nor do we equate it simply with a pedestrian accessibility indicator. Instead, it is treated as a proxy variable for public transport access conditions, used to characterize the convenience of accessing public transportation services in the vicinity of the residence (**see Lines448–463 in manuscript**):

It should be noted that in commuter rail or suburban railway systems, travelers often rely on multiple access modes for first- and last-mile connections (e.g., buses, cycling, and other non-motorized modes), rather than walking alone. This indicates a higher sensitivity to public transport connection conditions than in urban metro systems. For example, in a study of suburban rail commuting in Dhaka by Rahman[59] , it was found that multimodal combinations are widely used for first- and last-mile access. Therefore, the distance to the nearest bus stop effectively serves as a proxy for the adequacy of public transport feeder connections around Loop Line Railway stations. Given the relatively large station spacing and wide service coverage of the Loop Line Railway, residents tend to rely on buses and other public transport modes to complete first- and last-mile trips, with the bus system playing an important feeder and distribution role. Consequently, proximity to a bus stop correlates with reduced time costs and uncertainty associated with accessing the Loop Line Railway during commuting. This facilitates the perception and realization of the railway’s advantage as a “rapid commuting” mode, thereby enhancing its attractiveness as a commuting option.

Therefore, the mechanism of the "Distance to Nearest Bus Stop" variable in this study is not intended to characterize the walking environment itself. Instead, it indirectly affects the willingness to commute via the Loop Line Railway by influencing the time costs and uncertainties residents face when accessing the public transportation system during their commute. We have explicitly defined the meaning and interpretive boundaries of this variable within the text and have avoided over-interpreting it as direct evidence of actual transfer behavior.

## **Reviewers comment 8**

**It is unclear what Δy values refer to.**

## **Response to comment 8**

We appreciate the reviewer for pointing out the lack of clarity in the definition of $\text{Δy}$. We agree with this comment and have provided a clear explanation of the meaning and calculation method of Δy in the revised manuscript. Specifically, $\text{Δ}\text{y}^{\text{n}}$ represents the maximum magnitude of change in the model's predicted value for the n-th sample along the ICE curve as the target explanatory variable varies. It is used to measure whether the threshold effect of the built environment variable on an individual sample's commuting intention is significant. A larger $\text{Δ}\text{y}^{\text{n}}$ value indicates a stronger predictive impact of the variable on that sample, signifying a more pronounced threshold effect. (**see Lines 377-406 in manuscript**):

The Individual Conditional Expectation (ICE) model serves as a crucial interpretative tool for tree models. Compared with partial dependence plots (PDPs), which describe average effects, ICE plots visualize how the predicted outcome for each individual sample responds to changes in a target built-environment variable, thereby providing a more detailed representation of local heterogeneity within the sample set. However, the spatial characteristics of ICE results are often difficult to interpret directly[52]. Therefore, this study defines $\Delta y^{n}$as the variation in the predicted values along the ICE curve for the $n$-th sample to quantify the significance of threshold effects at the individual level. The calculation is expressed as follows:

$$\text{Δ}\text{y}^{\text{n}}\text{=max\{y(}\text{x}_{\text{z}}\text{,}\text{x}_{\text{c}}^{\text{(n)}}\text{)\}-min\{y(}\text{x}_{\text{z}}\text{,}\text{x}_{\text{c}}^{\text{(n)}}\text{)\}}$$

(6)

In equation (6), $\text{y(}\text{x}_{\text{z}}\text{,}\text{x}_{\text{c}}^{\text{(n)}}\text{)}$ denotes the predicted value for a given sample, $x_{z}$ represents the target explanatory variable, and $x_{c}^{n}$ refers to the observed values of the remaining variables for the $n$-th sample. The values of $x_{z}$ are varied across the full range of observed samples of the target explanatory variable. The indicator $\Delta y^{n}$captures the significance of the threshold effect: a larger value of $\Delta y^{n}$ indicates a more pronounced threshold effect of the built-environment factor on residents’ willingness to commute by the Loop Line Railway. Furthermore, the spatial distribution of $\Delta y^{n}$ reflects the spatial pattern of the strength of threshold effects associated with built environment factors.

On the basis of quantifying the significance of threshold effects, this study further defines“$m$”as the ratio of $\Delta y^{n}$ to $\Delta x_{z}^{n}$, where $\Delta x_{z}^{n}$ represents the variation in the sample value of the target explanatory variable corresponding to the maximum and minimum predicted values of sample “n”. The calculation is expressed as follows:

$$m=\frac{\text{Δ}\text{y}^{\text{n}}}{\text{Δx}_{z}^{n}}$$

(7)

In equation (7), the indicator “$m$” is derived from the ICE curves. A larger value of “$m$” indicates a stronger degree of non-linear influence of the built-environment factor on residents’ willingness to commute by the Loop Line Railway. Specifically, the “$m$” captures the non-linear effect of an individual built-environment variable on commuting willingness. By visualizing the spatial distribution of “$m$” , the spatial heterogeneity of such non-linear effects can be intuitively revealed. Furthermore, the spatial patterns of “$m$” provide a basis for examining the spatial non-stationarity in the dependence of commuting willingness on built-environment characteristics.

## **Reviewers comment 9**

**Results indicate that the effect of distance to city center is more pronounced in more remote areas, and suggests “improvements in distance conditions exert a substantial influence on residents' willingness to commute via the Loop Line Railway”. What improvements the authors refer to? This is very vague and needs specification. The same goes to the other three key variables.**

## **Response to comment 9**

We appreciate the reviewer for pointing out that the term "improvements" in the original manuscript was too vague. We agree that this wording could easily cause ambiguity, as it failed to specify what "improvement" refers to across different built environment variables. To address this issue, the revised manuscript clarifies each relevant expression. In the description of the results, we have avoided generalized terms like "improvements" and instead explicitly referred to the specific direction of change and the underlying mechanism of each variable. Specifically:

（1）In terms of population density, "improvement" does not simply refer to an increase in population size, but rather to the fact that, relative to lower-density suburban stations, central urban areas generate greater potential willingness through the clustering of population and employment. Consequently, the appeal of the Loop Line Railway as a commuting option is amplified for these residents.

（2）Regarding the distance to the nearest bus stop, "improvement" specifically involves measures in peripheral areas with limited bus resources, shortening the distance for residents to reach the nearest bus stop and reducing the time cost and uncertainty of the first- and last-mile can effectively enhance the commuting appeal of the Loop Line Railway.

（3）In terms of road network density, "improvement" refers to the fact that appropriately enhancing the connectivity and accessibility of the road network surrounding the station, increasing the number of alternative routes and interchange options available, will reduce barriers to residents commuting via the Loop Line Railway. This will consequently enhance commuting willingness for the Loop Line system.

（4）In terms of the distance to the city center, "improvement" does not refer to shortening the physical distance between residents' houses and the city center. Instead, it refers to the fact that the Loop Line Railway outperforms alternative modes in travel time, operating velocity, and cross-regional accessibility. Consequently, residents in remote locations can complete commutes within acceptable timeframes, thereby reinforcing their inclination to utilize the Loop Line Railway for daily travel.

The relevant expressions have been operationalized individually in the Results section of the revised manuscript (**see Lines 559–564, 581-584, 596-600, 611-615 in manuscript**). Furthermore, more standardized planning and policy implications have been consolidated within the Discussion and Recommendations section to avoid confusion between the interpretation of results and policy inferences.

## **Reviewers comment 10**

**It is unclear what m-values refer to.**

## **Response to comment 10**

We appreciate the reviewer for pointing out that the definition of the m-value was not sufficiently clear. We agree with this comment and have provided an explicit explanation of its meaning and calculation method in the revised manuscript. Specifically, the m-value is defined as the ratio of $\text{Δ}\text{y}^{\text{n}}$ to the corresponding change in the value of the target explanatory variable $\text{Δx}_{z}^{n}$. It is used to reflect the non-linear impact of an individual built environment variable on the willingness to commute via the Loop Line Railway; a larger value indicates a stronger degree of non-linear influence of the built environment variable on the commuting intention. For details, please see **Lines 395-406 in manuscript**).

## **Reviewers comment 11**

**There are some incomplete sentences, e.g., “In areas with lower bus stop density, establish industry development-oriented stations. Prioritise shuttle bus services or reduce transfer costs by increasing direct bus routes. Explore flexible transport options such as shared mobility to compensate for service gaps.” I won’t list all the examples, but this requires a careful proofread and revision**

## **Response to comment 11**

We appreciate the reviewer for pointing out this issue. We have re-examined the language and structure of the entire manuscript and confirmed that the original version indeed contained several incomplete sentences and that some suggestive statements were placed in inappropriate sections. In the revised manuscript, we have performed a systematic linguistic polishing and proofreading. We have removed incomplete or imprecise sentences from the Results and Analysis section and have unified and integrated the relevant suggestions into the "Recommendations" section for centralized elaboration. The issue of incomplete sentence structures has been resolved, and all relevant content has been standardized in terms of logic and semantics.

## **Reviewers comment 12**

**In Spatial Analysis of Threshold Effects, the results are largely mixed with policy implications. As such the section is unfocused and difficult to follow. Many of the policy implications are also broad-brush and overly comprehensive. Some of them are not necessarily supported by the analysis here. The authors should (1) discuss the key findings and (2) derive policy implications in a separate section. It should be noted that the implications should be firmly based on the empirical analysis. Normally 3-5 key implications would suffice. At the moment, the added values and implications from the study are difficult to discern and evaluate.**

## **Response to comment 12**

We appreciate the reviewer for the important comments regarding the structure and research contribution of this paper. We agree that the "Spatial Analysis of Threshold Effects" section in the original manuscript simultaneously presented empirical results and policy implications, which led to a lack of focus and hindered the clear communication of our findings and insights.

Following your suggestions, we have performed a systematic structural adjustment in the revised manuscript. We first summarize several key findings of this study and, based on these, refine policy implications that directly correspond to the empirical results. In the discussion of policy implications, we have removed broad recommendations that lacked direct empirical support, retaining only those conclusions clearly derived from the model results and threshold characteristics. This ensures logical consistency and traceability between the empirical analysis and policy insights. Through these revisions, the core findings, innovations, and practical significance of this research are now more centralized and transparent, making the contributions easier to assess. The specific details are as follows (**see Lines 646-723 in manuscript**):

Conclusions and Policy Recommendations

Conclusions

Based on survey data on residents’ willingness to commute via the Guiyang Loop Line Railway, this study systematically examines the non-linear effects of built environment factors on commuter willingness and their spatial heterogeneity. The main conclusions are as follows:

(1) Population density, distance to the nearest bus stop, road network density, and distance from the city center represent the four built environment factors with the most substantial impact on commuting willingness. Specifically, population density and road network density exert a positive influence, whereas distance to the nearest bus stop and distance from the city center show an inverse correlation with commuting propensity for the Loop Line Railway.

(2) The non-linear dependence of commuting willingness on the built environment exhibits strong spatial non-stationarity along the Loop Line Railway. The significance of threshold effects and the intensity of non-linear impacts associated with different built environment factors vary substantially across space, forming a clear gradient from the urban core to suburban and peripheral areas. Compared with the central urban area, residents living around suburban and peripheral stations show greater sensitivity to changes in feeder bus conditions, road network connectivity, and commuting distance, which results in more pronounced threshold effects and stronger non-linear responses.

(3) The mechanisms through which built environment factors influence commuting willingness differ across spatial contexts, with peripheral areas showing greater potential for improving commuting willingness. Specifically, the threshold effects and non-linear impacts of population density are primarily concentrated around central urban stations, indicating that in areas with highly concentrated commuting willingness, changes in population scale have stronger marginal effects on willingness to commute via the Loop Line Railway. In contrast, the non-linear effects of distance to the nearest bus stop and road network density are more pronounced around suburban and peripheral stations, indicating that in areas with relatively underdeveloped public transport and road infrastructure, first- and last-mile connection conditions and station accessibility represent key constraints on using the Loop Line Railway for commuting. In addition, the non-linear effect of distance to the city center is most pronounced at stations located farther from the urban core, suggesting that the Loop Line Railway has greater potential to mitigate the spatial disadvantage associated with long commuting distances in peripheral areas.

Policy Recommendations

Drawing on the spatial analysis of threshold effects and non-linear impacts of built environment factors, this study proposes the following recommendations to address the low ridership and underutilisation of the Guiyang Loop Line Railway, and to provide practical insights for station planning and the development of suburban railways in other cities.

(1) The results indicate that the commuting function of the Loop Line Railway depends heavily on the presence of stable and sufficiently concentrated commuting willingness. In areas with high population density and strong job–housing concentration, the Loop Line Railway is more suitable as a frequent and reliable commuting mode, and its commuter-oriented function should be prioritised. In contrast, in peripheral areas characterised by lower population density and dispersed residential patterns, changes in population scale alone are unlikely to meaningfully enhance the commuting attractiveness of the Loop Line Railway. In such contexts, improving feeder connections and reducing overall commuting time represent more effective strategies for strengthening its relative advantages.

(2) For suburban and peripheral stations, priority should be given to improving the accessibility of bus feeder services rather than expanding the overall scale of bus supply. Rather than increasing the number of bus routes, more effective measures include optimising the spatial distribution of bus stops, shortening walking distances to the nearest bus stop, and providing dedicated feeder bus services to railway stations. These measures can reduce first- and last-mile transfer costs and, in turn, enhance the commuting attractiveness of the Loop Line Railway.

(3) The model results show that the non-linear effects of road network density on commuting willingness are mainly concentrated in suburban and peripheral areas. Accordingly, road network improvements should focus on areas where transport infrastructure is relatively underdeveloped. Measures such as improving local street networks, reducing detour distances, and enhancing road connectivity can increase station accessibility and facilitate residents’ access to railway stations, thereby encouraging the use of the Loop Line Railway for commuting. In contrast, in central urban areas where road networks are already highly developed, further increases in road density may yield limited benefits. Instead, measures such as dedicated access lanes for railway transfers and enhanced traffic flow management can be adopted to reduce congestion and improve transfer efficiency.

(4) The Loop Line Railway plays an important role in mitigating the disadvantages associated with long commuting distances in peripheral areas. For residents living far from the urban core, commuting distance and time costs constitute major constraints on railway use. In these areas, the cross-regional rapid accessibility provided by the Loop Line Railway is more likely to be strongly perceived and valued. Accordingly, in the process of optimising urban spatial structure, the Loop Line Railway can serve as an important instrument for improving commuting conditions in peripheral areas and promoting shifts in commuting modes. However, given the relatively low level of development in these areas, particular attention should be paid to strengthening feeder transport facilities and reducing overall commuting time.

## **Reviewers comment 13**

**Figures are of very low resolutions.**

## **Response to comment 13**

We appreciate the reviewer for pointing out the insufficient resolution of the figures. In accordance with your comments, we have re-exported and replaced all images in the revised manuscript to enhance their resolution and clarity. The revised figures now ensure that all relevant information is clearly identifiable when magnified.

***Responses to comments from Reviewer #* 2**

## **Reviewers comment 1**

**The data are generally reliable, and the model estimation is reasonable. However, the manuscript still requires substantial improvements in terms of detailed descriptions of the data and methodological procedures. The language should also be carefully revised to improve clarity and meet to standard academic languages.**

## **Response to comment 1**

We appreciate the reviewer’s recognition of the data reliability and the reasonableness of our model estimation. In response to your suggestions for further improvement, we have systematically revised the manuscript in the following three aspects:

1. Enhancement of Data and Sample Descriptions In the methodology section of the revised manuscript, we have provided more granular details regarding the data sources and the survey process. This includes a more comprehensive and standardized description of the survey period, spatial scope, sampling methods, and sample size. Additionally, we have supplemented the rationale for selecting built environment variables and subjective perception variables, along with their definitions and measurement methods (**see Lines 259-356 in manuscript**).

2. Strengthening of Methodology and Modeling Procedures We have restructured and refined the explanation of the model construction and estimation process. We explicitly stated the theoretical basis for variable inclusion, interpreted the economic implications of key parameters, and provided supplementary details on the specific implementation steps for nonlinear effect identification and spatial heterogeneity analysis. These adjustments ensure a clearer and more complete methodological logic.

3. Comprehensive Revision of Language and Academic Expression The entire manuscript has undergone systematic proofreading and polishing. We focused on revising wordy expressions and imprecise concepts, standardized the use of key academic terminology, and strengthened the logical transitions between paragraphs to enhance the overall clarity and academic rigor of the paper.

***Responses to comments from Editor***

## **Editor’s comment 1**

**It is recommended that line numbers be added to the manuscript upon resubmission to make the review process more convenient and help provide more precise comments.**

## **Response to comment 1**

We sincerely appreciate the Editor’s valuable suggestion. Line numbers have been added throughout the revised manuscript to enhance readability and facilitate precise referencing and feedback during the review process.

## **Editor’s comment 2**

**The findings section of the abstract is overly generic. Although the study identifies several meaningful and distinctive patterns, the abstract merely states the existence of non-linear impacts and threshold effects. More specific and concrete findings should be highlighted to better reflect the study’s contribution.**

## **Response to comment 2**

We sincerely appreciate the Editor’s point regarding the lack of specificity in the research findings within the abstract. We have fully adopted this suggestion and systematically revised the abstract. The revised version moves beyond general statements like "nonlinear influences and threshold effects exist" to explicitly summarize the impacts of key built environment variables on the intention to commute via the Loop Line Railway. These revisions more accurately reflect the core findings and research contributions of this study. The specific modifications are as follows **(see Lines 16-37 in manuscript)**:

Abstract

This study takes the Guiyang Loop Line Railway as a case study and, based on resident commuting survey data, applies an XGBoost model combined with Individual Conditional Expectation (ICE) analysis to examine the threshold effects and non-linear spatial characteristics of the built environment on residents’ willingness to commute via the Loop Line Railway. The results indicate that: (1) population density, distance to the nearest bus stop, road network density, and distance to the city center are key built environment factors influencing commuting willingness. Among them, population density and road network density exhibit overall positive effects, whereas distance to the nearest bus stop and distance to the city center show negative effects; (2) the spatial distributions of the threshold effect magnitude (Δy) and the non-linear effect intensity (m) display a heterogeneous pattern broadly characterized by a “city center–suburban–peripheral” gradient, suggesting pronounced spatial non-stationarity in the relationship between the built environment and commuting willingness; (3) compared with the central urban area, residents living around suburban and peripheral stations are more sensitive to changes in bus feeder conditions, road network connectivity, and commuting distance, exhibiting stronger threshold effects and non-linear responses. These findings suggest that enhancing residents’ willingness to commute via the Loop Line Railway requires context-sensitive strategies that account for the built environment characteristics of different areas, with targeted improvements in bus–rail integration and road network conditions, rather than adopting uniform development and supporting policies across all stations。

## **Editor’s comment 3**

**The authors provide a detailed description of the metro system in Guiyang, which helps readers understand the background of the study area. However, the discussion of the key issues, such as “persistent issues of low utilization and sparse passenger numbers” and “core functions remain underutilized”, is insufficient. Providing empirical evidence (e.g., actual ridership statistics, service frequency data) or concrete examples would better justify the selection of the Guiyang Loop Line Railway as the study case.**

## **Response to comment 3**

We sincerely appreciate the Editor’s recognition of the study area background and the constructive suggestions regarding the justification for case selection. Addressing the insufficient elaboration on issues such as the "low utilization, sparse passenger flow, and underperformance of core functions" of the Guiyang Loop Line Railway, we have further supplemented and refined the Introduction section in accordance with your recommendations. The specific additions are as follows (**see Lines 127–144 in manuscript**):

Positioned as an important commuting corridor connecting multiple residential areas with the central urban area, the Loop Line Railway was designed to promote the development of the urban transport system, enhance connectivity between the city center and peripheral clusters, and support the construction of a “Guiyang–Gui’an one-hour commuting circle”. However, in comparison with its planned positioning and functional expectations, problems of low utilization and insufficient ridership have been faced by the Loop Line Railway in actual operation. Some studies indicate that since its opening, the average daily ridership of the entire line has been only about 3,000 passengers [3]. Media reports based on field observations at multiple stations further show that passenger volumes are limited during off-peak and most operating periods, with train occupancy rates remaining very low, and seat vacancy rates approaching 90% at certain times[17]. These findings indicate that the actual utilization of the Loop Line Railway falls short of its expected commuting service performance. This not only implies a failure of public investment to generate adequate social benefits but also suggests a weakening of the role of suburban railways in alleviating traffic congestion and promoting cross-regional commuting, thereby hindering improvements in urban traffic conditions and residents’ travel experience.

## **Editor’s comment 4**

**On pages 4–5, several factors contributing to the low utilization of the Loop Line Railway are listed (e.g., infrequent services, inadequate infrastructure, cumbersome transfers, and costs). However, built environment factors are barely discussed. Given that the core focus of the study is effects of the built environment on commuter willingness, the authors should clearly explain why built environment characteristics are particularly relevant in explaining low ridership. Strengthening this conceptual linkage would enhance the necessity of the study.**

## **Response to comment 4**

We sincerely appreciate the Editor’s significant comment. We agree that focusing solely on operational or institutional factors, such as ticket prices and transfer inconveniences, is insufficient to fully reveal the underlying mechanisms behind the limited commuting functionality of the Loop Line Railway. Furthermore, such a narrow focus fails to highlight the critical impact of the built environment on commuting intentions. Following your suggestions, we have supplemented and restructured the relevant content in the Introduction. By analyzing the current gaps in research regarding suburban railway commuting intentions, we have introduced the pivotal role played by the built environment in shaping residents' travel choices. The following content has been added to the revised manuscript (**see Lines 88–108 in manuscript**):：

for suburban railways characterized by larger station spacing and broader service catchments, the conversion of commuting willingness into actual ridership is not only dependent on train operating services but is also strongly constrained by the built environment conditions surrounding residents’ places of dwelling. In contrast to urban metro systems, a higher sensitivity to first- and last-mile travel conditions is exhibited by commuting behavior on suburban railways. When feeder bus services are insufficient, road network connectivity is poor, or station accessibility is low, the adoption of suburban railways as a regular commuting mode may be found difficult by residents, even when latent commuting willingness exists. Consequently, persistently low utilization of such lines is often observed. Nevertheless, operational and service supply factors have been the primary focus of existing studies on insufficient ridership, while limited attention has been paid to the mechanisms through which commuting willingness is influenced by the built environment, ultimately leading to low passenger volumes. On the one hand, a station-area (destination-end) perspective has been primarily adopted in prior research, emphasizing land use patterns or development intensity around stations; conversely, systematic empirical evidence regarding the effect of residential-end built environment characteristics on commuters’ mode choice remains scarce[13]. On the other hand, most existing studies are based on large cities with relatively simple topographic conditions[14]. Mountainous cities, where urban spatial structure is strongly constrained by natural terrain, have received far less attention, and the applicability of existing findings to such contexts has yet to be adequately examined. Based on this, re-examining the issue of low utilization rates for the Loop Line Railway from a built environment perspective holds clear theoretical necessity and practical significance.

## **Editor’s comment 5**

**Although commuter willingness is the central dependent variable, the literature review does not sufficiently cover previous studies on commuter willingness, including relevant theoretical frameworks and methodological approaches.**

## **Response to comment 5**

We sincerely appreciate the Editor’s valuable comments. We agree that as the core dependent variable of this study, it is essential to systematically review the theoretical foundation and research paradigm of commuting intention in the literature review. In response to this suggestion, we have added a "Literature Review" section to the revised manuscript and significantly expanded its content, focusing on the theoretical framework, research subjects, and methodological approaches related to commuting intention. Specifically:

We systematically reviewed the theoretical frameworks commonly used in commuting intention research, including the Theory of Planned Behavior (TPB) and the Technology Acceptance Model (TAM), and summarized the progress of related studies in explaining residents' intentions to choose public transport and commuting modes. In light of the long-distance commuting characteristics of suburban railways, we synthesized empirical findings from existing research regarding commuting intentions and their influencing factors on regional/suburban railways. Building upon this, we further identified the research gaps concerning the impact of built environment factors on commuting intention, clarifying the research positioning and innovation of this study: analyzing the influence of the built environment on suburban railway commuting intention from a residential-end perspective. The relevant content has been supplemented and refined in the "Literature Review" section of the revised manuscript (**see Lines 161–256 in manuscript**)：

Literature Review

In travel behavior research, the Theory of Planned Behavior (TPB) and the Technology Acceptance Model (TAM) have been widely utilized to examine individuals’ travel intentions. Proposed by Ajzen, it is posited by TPB that travel intention is jointly determined by attitude, subjective norms, and perceived behavioral control [18]. Extensive application of this framework has been observed in transportation studies to explain travel mode choice. It is suggested by empirical evidence that public transport use is significantly influenced by these three components, with attitude often identified as the most influential factor shaping travel decisions [19-21]. The critical roles of attitude and perceived behavioral control in determining public transport usage intention are further highlighted by other studies[22-23].

In the Technology Acceptance Model (TAM), originally developed by Davis [24], perceived usefulness and perceived ease of use are emphasized as key determinants of individuals’ acceptance of new technologies. With the emergence of new transport modes, increasing adoption of TAM has been witnessed in transportation research, including studies on electric vehicle adoption and new energy bicycles[25-26].

To provide a more comprehensive behavioral explanation, TPB and TAM have been integrated by some studies. For example, TPB, TAM, and habit theory were combined by Chen[27] to analyze private car users’ willingness to shift to public transport. Similarly, an integrated TPB-TAM framework was employed by Wang[28] ; it was found that perceived usefulness, perceived ease of use, attitude, subjective norms, and perceived behavioral control jointly influence individuals’ willingness to adopt sustainable travel modes.

Commuting is defined as the long-term, regular, and repetitive round-trip travel between individuals’ places of residence and employment, characterized by high regularity, persistence, and temporal rigidity[29]. With rapid economic development and ongoing urbanization, outward expansion of urban spaces continues, significantly increasing the distance between workplaces and residences as well as commuting times. Sustainable urban development is constrained by the resulting issues of traffic congestion, reduced travel efficiency, and diminished commuter satisfaction[30]. Owing to their high operating speed, large capacity, and long service distance, suburban railways are widely regarded as critical infrastructure for supporting medium- and long-distance commuting and for facilitating “one-hour commuting circles” in metropolitan regions [31]. In Europe, North America, and East Asia, suburban rail systems were developed relatively early, and mature operational frameworks have been achieved, exemplified by the Paris suburban railway network[32], the Tokyo commuter rail system[33], and the commuter rail systems in the United States[34]. Commuting willingness structures, passenger flow characteristics, and operational efficiency have been the primary focus of existing studies in these contexts, offering valuable insights into the role of suburban railways in metropolitan commuting systems. In contrast, an exploratory and developmental stage characterizes suburban railways in China. Although several lines - such as the Shanghai R Line[35] and the Beijing S Line[36] have been commissioned, unsatisfactory commuting ridership performance has generally been observed, and intended commuting functions have not been fully realized. This outcome has been primarily attributed by previous studies to factors related to commuter behavior and operational service attributes. For example, limitations in route alignment, pricing, and operational flexibility were identified by Alimo as major contributors to low suburban rail ridership[12]. Irawan found that walking distance, waiting time, and parking costs significantly influence commuters’ mode choice decisions[37]. Similar conclusions have been drawn in studies within China, which highlight service frequency, travel time[10], travel cost[38], infrastructure conditions, and development strategies[11] as key determinants of suburban rail usage. Beyond train operations and fare policies, the importance of commuting trip chains and access–egress conditions has begun to be emphasized in recent research. Brohi, drawing on the Theory of Planned Behavior, demonstrated that commuters’ attitudes toward public transport significantly influence their willingness to use suburban railways[39]. It was further suggested by Le that maintaining affordable fares while improving last-mile services—such as bike-sharing systems and feeder bus connections—can substantially enhance the commuting attractiveness of suburban railways [40]. Overall, although existing studies have provided a relatively comprehensive understanding of suburban railway commuting from the perspectives of commuting behavior, service quality, fare policy, and planning, most research remains focused on supply-side explanations or treats entire lines as the unit of analysis. As a result, the spatial determinants underlying variations in commuting performance across different stations along the same line remain insufficiently explored.

Against this backdrop, increasing attention has been paid to the influence of the built environment on residents’ commuting mode choice. Built environment characteristics are operationalized by most studies using the well-established “5D” framework—density, diversity, destination accessibility, design, and distance to transit—to evaluate their effects on travel behavior[41]. For example, Jia Fang, adopting the 5D perspective, demonstrated that built environment attributes such as block size and intersection density are strongly associated with residents’ mode choice decisions[42]. Wang examined the driving mechanisms of urban rail transit ridership and revealed non-linear relationships and interaction effects between built environment factors and ridership, thereby providing empirical support for rail transit planning[43]. Similarly, Yafei Xi further identified the spatiotemporal characteristics of non-linear relationships between metro station built environments and passenger flows, showing that factors such as population density and parking facility density exert significantly non-linear impacts on station-level ridership[44]. Although extensive research has confirmed that built environment attributes—such as population density and land-use mix—affect commuting mode choice by shaping travel distance, accessibility, and transfer convenience[45], existing studies have largely focused on urban rail transit and metro systems. Empirical evidence on suburban railways remains limited. Given their larger station spacing and broader service catchment areas, suburban railways place greater emphasis on access and egress conditions, making commuters more sensitive to variations in the surrounding built environment than in metro systems [46]. This further complicates the identification of non-linear behavioral responses and spatial differences in commuting willingness under diverse built environment contexts. While the examination of non-linear built environment effects on ridership and travel willingness has begun in a small number of recent studies, systematic analyses of spatial heterogeneity across different urban locations remain scarce. In summary, although the influence of the built environment on commuting behavior has been well established in urban rail transit research, a clear research gap remains for suburban railways—particularly regarding the non-linear effects of residential built environment characteristics on commuting willingness and their spatial heterogeneity. Addressing this gap is the core focus of the present study.

## **Editor’s comment 6**

**The description of the survey data is insufficiently detailed. Key information is missing, including the survey period, the format of the questionnaire, the measurement scale used for the items (e.g., five-point Likert scale), and whether the survey was conducted in paper-based or electronic form. If an online survey was used, the specific platform should also be stated.**

## **Response to comment 6**

This comment is similar to Comment 5 from Reviewer 1, and we have adopted the same response. In accordance with your suggestion, we have added a new section titled "Sample Selection and Survey Site Determination" in the revised manuscript to provide a detailed explanation. Please refer to **Lines 259–308 in manuscript**.

## **Editor’s comment 7**

**A total of 700 questionnaires were returned, but only 451 were used for model estimation, resulting in a valid response rate of 64.43%. The data cleaning process and exclusion criteria should be clearly described to ensure data processing is conducted rigorously.**

## **Response to comment 7**

We sincerely appreciate the Editor’s detailed feedback regarding the data processing procedures. We agree that a more transparent and standardized description of the questionnaire cleaning process and sample exclusion criteria is essential to ensure the rigor of the data treatment. Consequently, we have provided the following explanation in the revised manuscript (**see Lines 291-308 in manuscript**):

The primary survey was administered between April and May 2024 via face-to-face paper-based questionnaires distributed within a 3 km radius of each station. In total, 750 questionnaires were distributed. Following the exclusion of questionnaires with incomplete responses or significant logical inconsistencies, 700 valid questionnaires were retained. During the model estimation phase, a secondary screening of the sample was performed to ensure the analysis adequately captured the differential effects of the built environment on commuting willingness across varied spatial contexts. Specifically, a subset of 451 questionnaires was selected for analysis, drawn from six representative stations: Guiyang Station, Guiyang North Station, Baiyun North Station, Jinyang South Station, Gui’an Station, and Huaxi South Station. Substantial variations in built environment characteristics, spatial location, infrastructure provision, functional roles, and ridership levels are exhibited by these stations. Collectively, they represent core transportation hubs, regional sub-centers, and standard functional stations, reflecting a spatial gradient spanning from the urban center to suburban and peripheral zones. This sampling strategy facilitates a comprehensive examination of the heterogeneous effects of the built environment on individuals’ willingness to commute via the Loop Line Railway across diverse urban contexts.

## **Editor’s comment 8**

**Table 1 presents the measurement items for commuter willingness. However, it is unclear how these items were selected and on what basis. Additional explanations or supporting references should be provided.**

## **Response to comment 8**

We appreciate the Editor’s comments. As this suggestion is identical to Comment 6 from Reviewer 1, we have adopted the same response and incorporated the following content into the revised manuscript(**see Lines 162–183,310–343** **in manuscript**).

## **Editor’s comment 9**

**On page 14, please check the interpretation of the m-value in Equation (7). Specifically, it should be clarified whether a higher m-value indicates a greater degree of non-linearity in the effect or a greater overall magnitude of influence?**

## **Response to comment 9**

We appreciate the Editor’s correction. As this comment is identical to Comment 10 from Reviewer 1, we have adopted the same response and incorporated the following content into the revised manuscript **(see Lines 395-406 in manuscript**).

## **Editor’s comment 10**

**It is recommended that the authors include comparisons with findings from previous studies to better highlight the novel contributions of the current research.**

## **Response to comment 10**

We appreciate the Editor’s important suggestion regarding strengthening the comparison with existing studies. We have fully adopted this advice and, in the revised manuscript, have further clarified and reinforced the innovative contributions of this paper through direct comparisons with previous research findings.

Specifically, in the Literature Review and Conclusion sections, we systematically compared our results with existing findings on suburban railway and urban rail transit commuting behavior. Existing studies often focus on operational service levels, fares, or the built environment around stations (destination-end), typically assuming that built environment factors have a linear or spatially homogeneous impact on commuting behavior (**see Line 184-225 in manuscript**); furthermore, research subjects are primarily concentrated on urban subway systems or overall line-level analysis. In contrast, the empirical results of this study demonstrate that the impacts of key built environment factors—such as population density, bus feeder conditions, road network density, and distance to the city center—on regional railway commuting intention not only exhibit significant non-linear and threshold characteristics but also show pronounced spatial heterogeneity across different urban locations **(see Lines 423-483, 541-620 in manuscript)**. By systematically contrasting our findings with related research, the potential value of this study is better defined.

## **Editor’s comment 11**

**The first sentence on pages 14-15 is incomplete and should be revised for clarity.**

## **Response to comment 11**

Regarding the incomplete sentence on Pages 14–15: We appreciate the Editor’s correction. Regarding the incomplete first sentence on Pages 14–15, we have revised it to ensure the expression is clear and complete, thereby avoiding any potential ambiguity present in the original phrasing **(see Line 423-425 in manuscript)**: A relatively significant influence is exerted on commuting willingness by population density, distance to the nearest bus stop, road network density, and distance from the city center.

## **Editor’s comment 12**

**On page 17 The sentences beginning with “When built environment variables exhibit threshold effects…” is strikingly similar in wording to a Chinese-version publication. The authors should either explicitly cite the original source or rewrite the paragraph to avoid concerns regarding textual similarity.**

## **Response to comment 12**

Regarding the sentence on Page 17 starting with "When built environment variables exhibit threshold effects…": We appreciate the Editor’s correction. Regarding the sentence on Page 17 starting with "When built environment variables exhibit threshold effects…", we noted that the original phrasing showed high similarity to existing literature. We have entirely reformulated this paragraph and included explicit citations to the original sources where necessary to ensure academic integrity and originality **(see Line 484-490 in manuscript)**:

As illustrated in Fig. 3, although the four built environment categories exert differing degrees of influence on commuting willingness, all observations exhibit non-linear characteristics at the threshold, suggesting a consistent threshold effect across the entire dataset. When threshold effects are present, the magnitude of the change in predicted values (Δy) reflects the significance of the threshold effect for a specific observation. A larger (Δy) indicates a more pronounced threshold effect for the corresponding observation. The spatial distribution of (Δy) reveals the spatial heterogeneity in threshold-effect significance across various locations.

## **Editor’s comment 13**

**The statement on page 18, “This suggests that in peripheral urban areas, changes in population density have a relatively limited impact on commuting willingness, with a less pronounced threshold effect”, is not sufficiently precise. A weaker threshold effect does not necessarily imply a limited overall impact. Similar issues of imprecise appear elsewhere in the manuscript, requiring more careful wording to ensure conceptual and empirical accuracy.**

## **Response to comment 13**

Regarding the conceptual imprecision of "weak threshold effect": We appreciate the Editor for pointing out the lack of conceptual rigor in this expression. We agree that the original manuscript's phrasing—equating "a weak threshold effect" directly with "population density having a limited impact on commuting intention"—was logically imprecise. It conflated the prominence of the variable's threshold characteristics with its overall impact on commuting intention. Following your suggestion, we have reformulated the relevant paragraphs as follows. It should be noted that the reduction in the significance of the threshold effect does not imply that population density has no impact on commuting intention. Instead, it indicates that in peripheral areas where population density is generally low and residential distribution is relatively dispersed, the impact of population scale on commuting intention is more likely to manifest as a relatively gradual or quasi-linear response, rather than abrupt leaps within specific intervals. This result reflects the spatial heterogeneity in the underlying mechanisms through which population density operates across different urban locations **(see Line 496-507 in manuscript)**.

Fig. 4a indicates that (Δy) values for population density exhibit a decreasing spatial gradient from the city center to suburban and peripheral regions. High (Δy) values are concentrated near Guiyang Station and Guiyang North Station; conversely, high-value clusters are less prevalent in suburban locations (e.g., Baiyun North and Jinyang South stations) and peripheral areas (e.g., Huaxi South and Gui’an stations), where the distribution of (Δy) appears more localized. This pattern is driven by the high population density and functional agglomeration of central urban areas, where changes in population scale trigger concurrent variations in commuting willingness and travel pressure, thereby accentuating threshold effects. In contrast, lower population density and dispersed residential development in peripheral areas result in a smaller marginal influence of population variations on commuting decisions, leading to less pronounced threshold effects.

## **Editor’s comment 14**

**The section title “Spatial Analysis of Threshold Effects” does not accurately reflect the content of the section, which primarily discusses non-linear impacts rather than threshold effects.**

## **Response to comment 14**

We sincerely appreciate the Editor’s valuable feedback regarding the chapter titles. We acknowledge that the error was due to an oversight in the writing process, which resulted in the incorrect titling of these sections. We have fully adopted your suggestion and revised the chapter titles to “Spatial Characteristics of Non-Linear Effects”, ensuring they more accurately reflect the content of the manuscript **(see Line 540 in manuscript**). We are grateful for your correction and sincerely apologize for this carelessness.。

## **Editor’s comment 15**

**The manuscript lacks a discussion of limitations and future research directions, which should be added.**

## **Response to comment 15**

We appreciate the Editor’s suggestion. The original manuscript did not include a discussion on the research limitations and future research directions. To improve the content of the paper, we have added a dedicated section in the revised manuscript. The specific details are as follows **(see Lines 621-644 in manuscript**):

Limitations and Future Research Directions

Although this study provides a systematic analysis of the non-linear effects and spatial heterogeneity of built environment factors on commuting willingness, several limitations remain that warrant further investigation.

First, this research is limited to the Guiyang Loop Line Railway as a single case studay. As a typical mountainous city, Guiyang possesses distinctive topographical features, urban spatial structures, and a unique rail transit development stage. Therefore, caution is required when generalizing the findings to cities with divergent geographical conditions or development trajectories. Future studies should incorporate comparative analyses across multiple cities or railway lines to validate the robustness and generalizability of the conclusions.

Second, the analysis relies on cross-sectional survey data, which captures residents’ stated commuting willingness at a single temporal point. Consequently, the study cannot fully capture dynamic shifts in commuting willingness resulting from built environment improvements or operational adjustments. Future research could benefit from integrating longitudinal survey data with objective travel data (e.g., smart card or mobile phone signaling data) to better examine the long-term behavioral impacts of built environment interventions.

Finally, data constraints limited the built environment measurement to indicators such as population density, road network density, and bus stop proximity. Other potentially relevant factors, such as pedestrian environment quality and public transport reliability, were not explicitly considered. Future studies should incorporate finer-grained spatial and environmental data to elucidate the mechanisms through which the built environment influences suburban railway commuting behavior.

## **Editor’s comment 16**

**The manuscript structure is incomplete and does not fully conform to the conventions of academic writing in international journals. For example, the paper lacks: a standalone literature review section that clearly identifies research gaps, a detailed description of the questionnaire design and data collection procedures, and a section discussing limitations and directions for future research.**

## **Response to comment 16**

We appreciate the Editor’s meticulous review of the manuscript’s structure. We agree that the original version had structural deficiencies, such as the absence of a dedicated literature review to clarify research gaps, a detailed description of the questionnaire design and data collection procedures, and a section discussing research limitations and future directions. In the revised manuscript, we have added a literature review section, provided a detailed explanation of the questionnaire collection process, and discussed the study's limitations and future research directions. Please refer to **Lines 161-256 ,621-644in the revised manuscript** for details.

## **Editor’s comment 17**

**Although the language is generally concise, the manuscript contains multiple informal expressions and instances of Chinglish. In addition, many sentences are low in information density and overly generic (e.g., “The questionnaire content is as shown in Table 1” or repeated vague expressions like “However, indiscriminately increasing… may/could…”). The language should be carefully revised to improve precision.**

## **Response to comment 17**

We appreciate the Editor’s meticulous review. We agree that the original manuscript contained some informal expressions, "Chinglish," and instances of low information density, overly general phrasing, and repetitive sentences. In the revised manuscript, we have conducted a systematic linguistic polishing of the entire text. The primary improvements include:

Refining Generalizations: Vague or repetitive phrasing has been rewritten into specific and precise academic language.

Standardizing Academic Tone: We have performed a comprehensive correction of grammar, sentence structures, and vocabulary to ensure the language meets the standards of international academic journals.

Restructuring for Clarity: Long or fragmented sentences have been reorganized into clear, well-structured academic expressions to improve readability and flow.

***Responses to*** ***to Journal Requirements***

## **comment 1**

Please ensure that your manuscript meets PLOS ONE's style requirements, including those for file naming. The PLOS ONE style templates can be found at https://journals.plos.org/plosone/s/file?id=wjVg/PLOSOne_formatting_sample_main_body.pdfandhttps://journals.plos.org/plosone/s/file?id=ba62/PLOSOne_formatting_sample_title_authors_affiliations.pdf

## **Response to comment 1**

We have carefully reviewed the PLOS ONE style requirements and the provided templates. We have updated the manuscript's formatting, including the title page, heading styles, and reference citations, to ensure full compliance with the journal's standards. Furthermore, we have checked and adjusted the file naming of all submitted documents as per the instructions.

## **comment 2**

Please provide additional details regarding participant consent. In the ethics statement in the Methods and online submission information, please ensure that you have specified (1) whether consent was informed and (2) what type you obtained (for instance, written or verbal, and if verbal, how it was documented and witnessed). If your study included minors, state whether you obtained consent from parents or guardians. If the need for consent was waived by the ethics committee, please include this information.

If you are reporting a retrospective study of medical records or archived samples, please ensure that you have discussed whether all data were fully anonymized before you accessed them and/or whether the IRB or ethics committee waived the requirement for informed consent. If patients provided informed written consent to have data from their medical records used in research, please include this information.

## **Response to comment 2**

For this study, verbal informed consent was obtained from all participants prior to the face-to-face interviews. Since the survey was conducted in a public area and did not involve any sensitive personal or medical data, it was considered a low-risk observational study. The verbal consent process was conducted as follows: Before each interview, the investigator read a standard introductory statement to the participant, explaining the study's purpose, the voluntary nature of participation, and the complete anonymity of the collected data. The participant's consent was witnessed and documented by the investigator by checking a specific "Consent Obtained" box on the first page of the questionnaire before proceeding. No data was recorded from individuals who did not provide this explicit verbal agreement.

## **comment 3**

Please note that PLOS One has specific guidelines on code sharing for submissions in which author-generated code underpins the findings in the manuscript. In these cases, we expect all author-generated code to be made available without restrictions upon publication of the work. Please review our guidelines at https://journals.plos.org/plosone/s/materials-and-software-sharing#loc-sharing-code and ensure that your code is shared in a way that follows best practice and facilitates reproducibility and reuse

## **Response to comment 3**

We have reviewed PLOS ONE’s guidelines on code sharing. To ensure reproducibility, the key parts of our author-generated code used for data analysis have been made available without restrictions. We have uploaded these scripts to gitHub, and they can be accessed via the following repository: https://github.com/noininoinoin/The-Loop-Line-Railway

## **comment 4**

In your Methods section, please provide additional information regarding the permits you obtained for the work. Please ensure you have included the full name of the authority that approved the field site access and, if no permits were required, a brief statement explaining why.

## **Response to comment 4**

For this study, no specific permits were required for the field site access because the questionnaire survey was conducted in open public spaces (such as station squares and public sidewalks) where no restricted access or institutional permission is needed for academic research purposes.

## **comment 5**

Please provide a complete Data Availability Statement in the submission form, ensuring you include all necessary access information or a reason for why you are unable to make your data freely accessible. If your research concerns only data provided within your submission, please write "All data are in the manuscript and/or supporting information files" as your Data Availability Statement.

## **Response to comment 5**

We have provided a complete Data Availability Statement in the online submission form. All relevant data underlying the findings of this study are fully available without restriction. The data have been deposited in the Figshare repository and can be accessed via the following DOI:https://10.6084/m9.figshare.31146256

## **comment 6**

 We note that you have indicated that there are restrictions to data sharing for this study. PLOS only allows data to be available upon request if there are legal or ethical restrictions on sharing data publicly. For more information on unacceptable data access restrictions, please see http://journals.plos.org/plosone/s/data-availability#loc-unacceptable-data-access-restrictions. Before we proceed with your manuscript, please address the following prompts: a) If there are ethical or legal restrictions on sharing a de-identified data set, please explain them in detail (e.g., data contain potentially identifying or sensitive patient information, data are owned by a third-party organization, etc.) and who has imposed them (e.g., a Research Ethics Committee or Institutional Review Board, etc.). Please also provide contact information for a data access committee, ethics committee, or other institutional body to which data requests may be sent. b) If there are no restrictions, please upload the minimal anonymized data set necessary to replicate your study findings to a stable, public repository and provide us with the relevant URLs, DOIs, or accession numbers. For a list of recommended repositories, please see https://journals.plos.org/plosone/s/recommended-repositories. You also have the option of uploading the data as Supporting Information files, but we would recommend depositing data directly to a data repository if possible. We will update your Data Availability statement on your behalf to reflect the information you provide.

## **Response to comment 6**

We clarify that there are no restrictions to data sharing for this study. All relevant data underlying the findings are fully available without restriction. The privacy information of the subjects has been anonymized and the dataset has been deposited in the Figshare repository.

## **comment 7**

Thank you for stating the following in the Acknowledgments Section of your manuscript:
The research is supported by the National Natural Science Foundation of China [Grant No. 71864008].The funder website is https://www.nsfc.gov.cn/. The funder  did not participate in the research design, data collection and analysis, publication decisions, or manuscript writing.

## **Response to comment 7**

We have confirmed that the funding information is correctly provided. As per PLOS ONE’s guidelines, we have ensured that this funding statement is included in the Financial Disclosure section of the online submission form. We also confirm that we have removed all funding-related information from the Acknowledgments section of the manuscript to avoid duplication, as required by the journal.

## **comment 8**

[Please update your submission to use the PLOS LaTeX template. The template and more information on our requirements for LaTeX submissions can be found at https://journals.plos.org/plosone/s/latex.](Please%20update%20your%20submission%20to%20use%20the%20PLOS%20LaTeX%20template.%20The%20template%20and%20more%20information%20on%20our%20requirements%20for%20LaTeX%20submissions%20can%20be%20found%20at%20https://journals.plos.org/plosone/s/latex.)

## **Response to comment 8**

We have updated the manuscript to strictly follow the official PLOS LaTeX template. We have ensured that the preamble, font settings, and section hierarchies are in full compliance with the journal's requirements as specified in the provided link.

## **comment 9**

 Please amend the manuscript submission data (via Edit Submission) to include author Xiaohuan Wang, Yahao Guo, Xiwei Xiong.

## **Response to comment 9**

We have amended the manuscript submission data as requested, the author list remains unchanged. The current authorship accurately reflects all contributors who meet the authorship criteria.

## **comment 10**

We note that Figure(s) 1, 2, 4, 5, 6, 7, and 8, in your submission contain [map/satellite] images which may be copyrighted. All PLOS content is published under the Creative Commons Attribution License (CC BY 4.0), which means that the manuscript, images, and Supporting Information files will be freely available online, and any third party is permitted to access, download, copy, distribute, and use these materials in any way, even commercially, with proper attribution. For these reasons, we cannot publish previously copyrighted maps or satellite images created using proprietary data, such as Google software (Google Maps, Street View, and Earth). For more information, see our copyright guidelines: <http://journals.plos.org/plosone/s/licenses-and-copyright>...

## **Response to comment 10**

 We clarify that Figures 1, 2, 4, 5, 6, 7, and 8 were originally created by the authors for this study and do not contain any copyrighted satellite or proprietary images. The base map data was obtained from https://www.gscloud.cn, which is made available under the Open Database License (ODbL). We have updated the figure captions to explicitly state the data source.

## **comment 11**

Please ensure that you refer to Figure 1 in your text as, if accepted, production will need this reference to link the reader to the figure.

## **Response to comment 11**

We have ensured that Figure 1 is properly cited in the main text of the revised manuscript. We have checked all other figures (Figures 2–8) to ensure they are also correctly referenced in the text to facilitate the production process.

## **comment 12**

If the reviewer comments include a recommendation to cite specific previously published works, please review and evaluate these publications to determine whether they are relevant and should be cited. There is no requirement to cite these works unless the editor has indicated otherwise.

## **Response to comment 12**

We have carefully reviewed and evaluated the specific publications recommended by the reviewers. We agree that these works are highly relevant to our study on the Loop Line Railway and have integrated the necessary citations into the revised manuscript to provide a more comprehensive context for our findings.
