# Supplementary material for: Spatial heterogeneity of the nonlinear impact of built environment on suburban railway commuting willingness: Taking Guiyang Loop Line Railway as an example
Source: PLoS One. 2026 Apr 7;21(4):e0346358. doi: 10.1371/journal.pone.0346358 (PMC13056205; doi:10.1371/journal.pone.0346358)
Supplement: Attachment — Submitted filename: Response_to_Reviewers_auresp_2.docx [file pone.0346358.s004.docx]

**Point-to-Point Responses to Reviewers**

The authors would like to sincerely thank the Editor and the reviewers for their continued time and constructive comments on the revised manuscript. We greatly appreciate the valuable suggestions provided during this second round of review, which have helped us further improve the quality and clarity of the paper. We have carefully considered all comments and revised the manuscript accordingly. A detailed, point-by-point response is provided below, and all corresponding revisions have been clearly indicated in the manuscript.

***Responses to comments from Reviewer #* 1**

## **Reviewers comment 1**

**While the authors have largely revised the introduction. Certain parts can still be improved. Specifically, the authors elaborated extensively on the case of Guiyang in terms of suburban railway development and its impact on commuting, this is still case-specific and does not really help highlight the research contribution and position the study in the literature. I would suggest move more case-specific stuff to the study context in methodology and clearly argue the contribution of the study.**

## **Response to comment 1**

Thank you for this valuable suggestion. We agree that the previous version of the Introduction contained excessive case-specific descriptions related to Guiyang, which may have weakened the clarity of the research contribution and its positioning within the broader literature. Following the reviewer’s recommendation, we have substantially revised the Introduction section. Specifically, detailed descriptions of the Guiyang suburban railway development context have been reduced and relocated to the Study Area subsection in the Research Design. The Introduction now focuses more explicitly on the research gaps in existing literature, particularly regarding the non-linear effects and spatial heterogeneity of built environment factors on suburban railway commuting behavior. In addition, we have clarified the academic contributions of this study by emphasizing three aspects: (1) integrating machine learning approaches with behavioral perspectives to analyze commuting willingness, (2) identifying threshold effects of built environment factors, and (3) revealing spatial heterogeneity in suburban railway contexts within mountainous cities. These revisions improve the theoretical positioning and highlight the novelty of the study within the existing literature. **The revised text are as follows (see Lines 1–65 in manuscript)**

**Introduction**

Suburban railways, often termed commuter rail or suburban rail, are categorized as a form of passenger rail system positioned between urban rail transit (e.g., metro systems) and intercity railways; they are characterized by commuter-oriented services, relatively high operating speeds, and large transport capacity. As a long-distance commuting mode, a crucial role is played by suburban railways in connecting central urban areas with peripheral suburbs and in supporting the development of a “one-hour commuting circle”[1]. In comparison to conventional urban rail transit, a wider spatial area is typically served by suburban railways, which feature larger station spacing, higher operating speeds, and lower service frequencies. They are primarily designed to accommodate medium-to long-distance commuting between central cities and surrounding suburban areas or satellite towns. Their functional orientation is focused on establishing efficient commuting corridors within metropolitan area[2-3]. In this context, a significant contribution is made by suburban railways toward relieving functional pressure in central urban areas, shaping rational commuting structures, facilitating convenient daily mobility, and improving accessibility in peripheral urban regions[4].

With the continuous expansion of urban space and the deepening spatial mismatch between residential and employment locations, increasing scholarly attention has been attracted by suburban railways worldwide. The potential role of suburban rail systems in supporting metropolitan commuting structures has been highlighted by existing studies, viewed from perspectives such as urban land-use optimization, spatial structure refinement, and regional integration[5-6]. However, within the Chinese context, intended commuting functions have not been fully realized by many operational suburban railway lines, which commonly face problems of low ridership and underutilization [7-8]. To explain these phenomena, the limited commuting performance of suburban railways has been mainly attributed by previous studies to operational or institutional factors, including inappropriate station location, relatively high fares, long waiting times, and insufficient supporting infrastructure [9-11].

However, for suburban railways characterized by larger station spacing and broader service catchments, the conversion of commuting willingness into actual ridership is not only dependent on train operating services but is also strongly constrained by the built environment conditions surrounding residents’ places of dwelling. In contrast to urban metro systems, a higher sensitivity to first- and last-mile travel conditions is exhibited by commuting behavior on suburban railways. When feeder bus services are insufficient, road network connectivity is poor, or station accessibility is low, the adoption of suburban railways as a regular commuting mode may be found difficult by residents, even when latent commuting willingness exists. Consequently, persistently low utilization of such lines is often observed. Nevertheless, operational and service supply factors have been the primary focus of existing studies on insufficient ridership, while limited attention has been paid to the mechanisms through which commuting willingness is influenced by the built environment, ultimately leading to low passenger volumes. On the one hand, a station-area (destination-end) perspective has been primarily adopted in prior research, emphasizing land use patterns or development intensity around stations; conversely, systematic empirical evidence regarding the effect of residential-end built environment characteristics on commuters’ mode choice remains scarce[12]. On the other hand, most existing studies are based on large cities with relatively simple topographic conditions[13], Mountainous cities, where urban spatial structure is strongly constrained by natural terrain, have received far less attention, and the applicability of existing findings to such contexts has yet to be adequately examined.

To address these research gaps, this study investigates how residential built environment characteristics influence commuters’ willingness to use suburban railways, using Guiyang, China—a typical mountainous city—as an empirical case. Specifically, this study aims to: (1) examine the nonlinear relationships between residential built environment factors and suburban rail commuting willingness; (2) identify threshold effects and spatial heterogeneity of key influencing variables using machine learning approaches; and (3) provide planning implications for improving suburban railway utilization in mountainous cities. This study contributes to the existing literature in three aspects. First, it shifts the analytical perspective from station-area characteristics to residential-side built environment factors influencing suburban rail commuting behavior. Second, it extends suburban rail research to mountainous urban contexts where terrain constraints play an important role. Third, it applies data-driven methods to reveal nonlinear and heterogeneous mechanisms underlying suburban rail use, thereby providing new insights into improving suburban railway performance.

## **Reviewers comment 2**

**The logic flow of literature review is still very unclear and requires re-organization. It taps on a suite of relevant topics including TPB, commuting, suburban railway, and in certain parts, mixed stuff. I would urge the authors to more carefully organize the topics into sub-sections with leading sentences clearly showing the logic flow across different but related topics, and last, clearly and convincingly justify the research gaps to be addressed.**

## **Response to comment 2**

Thank you very much for this insightful and constructive comment. We fully agree that a clear logical structure is essential for presenting the literature review and positioning the research contribution. Following the reviewer’s suggestion, the literature review has been substantially reorganized and improved in the revised manuscript. Specifically, the literature review is now structured into three thematic subsections with clearer logical progression:

Behavioral Theories and Commuting Intention — This subsection introduces the theoretical foundations, including the Theory of Planned Behavior (TPB) and the Technology Acceptance Model (TAM), to explain the psychological mechanisms underlying commuting intention.

Suburban Railways and Commuting Research — This subsection reviews existing studies on suburban railway systems and commuting characteristics, highlighting current research focuses and limitations, particularly the dominance of operational and supply-side explanations.

Built Environment Effects and Research Gaps — This subsection synthesizes research on built environment influences on travel behavior, identifies the limited evidence in suburban railway contexts, and clearly articulates the research gaps related to non-linear effects and spatial heterogeneity.

The research gaps are now more clearly justified, emphasizing the need to examine the non-linear and spatially heterogeneous effects of residential built environment factors on suburban railway commuting willingness. **The revised text are as follows (see Lines 66–163 in manuscript)**

**Literature Review**

**Behavioral Theories and Commuting Intention**

In travel behavior research, behavioral theories have been widely applied to explain individuals’ travel intentions and mode choice decisions. Among them, the Theory of Planned Behavior (TPB) and the Technology Acceptance Model (TAM) are two of the most commonly adopted theoretical frameworks. According to the Theory of Planned Behavior proposed by Ajzen, individuals’ behavioral intentions are jointly determined by attitude, subjective norms, and perceived behavioral control [14]. This framework has been extensively applied in transportation studies to explain travel mode choice behavior. Empirical evidence suggests that public transport use is significantly influenced by these three components, with attitude often identified as the most influential factor shaping travel decisions[15-17]. Other studies further highlight the critical roles of attitude and perceived behavioral control in determining individuals’ intentions to use public transportation[18-19]. The Technology Acceptance Model (TAM), originally developed by Davis[20] , emphasizes perceived usefulness and perceived ease of use as key determinants influencing individuals’ acceptance of new technologies. With the emergence of new transportation modes, TAM has increasingly been applied in transportation research, including studies on electric vehicle adoption and new energy bicycle usage[21-22]. To provide more comprehensive behavioral explanations, some studies have integrated TPB and TAM frameworks. For example, Chen combined TPB, TAM, and habit theory to analyze private car users’ willingness to shift to public transport[23]. Similarly, Wang employed an integrated TPB–TAM framework and found that perceived usefulness, perceived ease of use, attitude, subjective norms, and perceived behavioral control jointly influence individuals’ willingness to adopt sustainable travel modes[24] .These studies provide an important theoretical foundation for examining commuting willingness in emerging transportation contexts.

**Suburban Railways and Commuting Research**

Commuting is defined as the long-term, regular, and repetitive round-trip travel between individuals’ places of residence and employment, characterized by high regularity, persistence, and temporal rigidity[25]. With rapid economic development and continuous urban expansion, the spatial separation between workplaces and residences has increased significantly, resulting in longer commuting distances and travel times. These changes contribute to traffic congestion, reduced travel efficiency, and decreased commuter satisfaction, thereby constraining sustainable urban development[26]. Due to their high operating speed, large passenger capacity, and long service distance, suburban railways are widely regarded as critical infrastructure for supporting medium- and long-distance commuting and for facilitating “one-hour commuting circles” in metropolitan regions[27]. In Europe, North America, and East Asia, suburban rail systems were developed relatively early and have achieved relatively mature operational frameworks, as exemplified by the Paris suburban railway network[28] , the Tokyo commuter rail system[29] , and commuter rail systems in the United States[30] . Existing studies in these contexts primarily focus on commuting demand structures, passenger flow characteristics, and operational efficiency, providing valuable insights into the role of suburban railways in metropolitan commuting systems.

In contrast, suburban railways in China remain in an exploratory and developmental stage. Although several lines, such as the Shanghai R Line[31] and the Beijing S Line[32] , have been commissioned, commuting ridership performance has generally been unsatisfactory, and intended commuting functions have not been fully realized. Previous studies have largely attributed this outcome to factors related to commuter behavior and operational service attributes. For instance, limitations in route alignment, pricing, and operational flexibility were identified by Alimo as major contributors to low suburban rail ridership[11] . Irawan found that walking distance, waiting time, and parking costs significantly influence commuters’ mode choice decisions[33]. Similar conclusions have been drawn in studies conducted in China, which highlight service frequency, travel time[9] ,travel cost[34], infrastructure conditions, and development strategies[10] as key determinants of suburban rail usage.

Beyond operational attributes and fare policies, recent research has begun to emphasize the importance of commuting trip chains and access–egress conditions. Brohi, drawing on the Theory of Planned Behavior, demonstrated that commuters’ attitudes toward public transport significantly influence their willingness to use suburban railways[35]. Le further suggested that maintaining affordable fares while improving last-mile services—such as bike-sharing systems and feeder bus connections—can substantially enhance the commuting attractiveness of suburban railways[36]. Overall, although existing studies have provided a relatively comprehensive understanding of suburban railway commuting from behavioral and operational perspectives, most research remains focused on supply-side explanations or treats entire railway lines as the unit of analysis.

**Built Environment Effects and Research Gaps**

Against this backdrop, increasing attention has been paid to the influence of the built environment on residents’ commuting mode choice. Built environment characteristics are commonly operationalized using the well-established “5D” framework—density, diversity, destination accessibility, design, and distance to transit—to evaluate their effects on travel behavior[37]. For example, Jia Fang demonstrated that built environment attributes such as block size and intersection density are strongly associated with residents’ mode choice decisions[38]. Wang examined the driving mechanisms of urban rail transit ridership and revealed non-linear relationships and interaction effects between built environment factors and ridership, thereby providing empirical support for rail transit planning[39]. Similarly, Yafei Xi identified spatiotemporal non-linear relationships between metro station built environments and passenger flows, showing that factors such as population density and parking facility density exert significantly non-linear impacts on station-level ridership[40].

Although extensive research has confirmed that built environment attributes—such as population density and land-use mix—affect commuting mode choice by shaping travel distance, accessibility, and transfer convenience[41] , most existing studies focus on urban rail transit and metro systems. Empirical evidence on suburban railways remains limited. Compared with metro systems, suburban railways typically have larger station spacing and broader service catchment areas, placing greater emphasis on access and egress conditions. As a result, commuters may be more sensitive to variations in the surrounding built environment in suburban railway contexts[42]. This further complicates the identification of non-linear behavioral responses and spatial differences in commuting willingness across different urban locations.

While a small number of recent studies have begun to examine non-linear built environment effects on ridership and travel willingness, systematic analyses of spatial heterogeneity across stations within the same suburban railway line remain scarce. Therefore, a clear research gap exists regarding how residential built environment characteristics influence suburban railway commuting willingness, particularly in terms of non-linear effects and spatial heterogeneity. Addressing this gap constitutes the core objective of the present study.

## **Reviewers comment 3**

**In section 3.1.1, a map should be provided to map out the survey stations and possibly the distribution of the sample to allow a more straightforward presentation of the survey outcome.**

## **Response to comment 3**

Thank you for this helpful suggestion. A map illustrating the survey stations has already been provided in Section 3.2 (Fig. 1). To improve clarity and ensure that the spatial distribution of the survey is more explicitly presented, we have further revised the figure caption and corresponding text to highlight the locations of the survey stations and the coverage of the samples. The relevant description has been updated accordingly in the revised manuscript. **see Line 212 in manuscript)**

Fig. 1 Schematic diagram of survey points and study area

*Note：*This figure was produced by the author using ArcGIS

## **Reviewers comment 4**

**Section 3.1.2, the theorical foundation can be underpinned by a conceptual framework.**

## **Response to comment 4**

Thank you for this valuable suggestion. We agree that presenting the theoretical foundation in a conceptual framework would help improve the clarity of the theoretical structure. Following the reviewer’s recommendation, a conceptual framework figure **(Fig. 2)** has been added in Section 3.3 to visually present the relationships among the key constructs derived from the Theory of Planned Behavior (TPB) and the Technology Acceptance Model (TAM). The purpose of this framework is to illustrate the psychological mechanisms underlying commuting intention formation, including perceived usefulness, perceived ease of use, attitude, subjective norms, and perceived behavioral control. Since the primary objective of this study is to examine the effects of built environment characteristics on commuting willingness using empirical modeling approaches, the conceptual framework focuses on the theoretical behavioral foundation rather than incorporating all empirical variables.We believe this addition improves the clarity of the theoretical basis while maintaining consistency with the research focus of the paper.

|  |  |
| --- | --- |
| a.the Theory of Planned Behavior | b.the Technology Acceptance Model |
| Fig. 2 Foundational Model of Travel Behaviour | |

## **Reviewers comment 5**

**Section 4.2, how was the level of significance of independent variables determined, since the XGBoost does not generate significance test?**

## **Response to comment 5**

Thank you very much for this insightful comment. We agree that XGBoost is a machine learning algorithm and does not provide statistical significance tests (e.g., p-values) in the same manner as traditional econometric models. In this study, the term “significant influence” does not refer to statistical significance in the inferential sense. Instead, it refers to the relative importance and contribution of explanatory variables to the prediction of commuting willingness, as quantified by model-based feature importance and SHAP value analysis.

Specifically, the independent variables included in Section 4.2 were selected based on their higher contribution scores derived from the XGBoost model, as well as their interpretability in the SHAP-based marginal effect analysis. The threshold effects discussed in this section were identified through the ICE (Individual Conditional Expectation) curves, which capture the non-linear response patterns of the model predictions to variations in each variable. Therefore, the “significance” mentioned in the manuscript reflects predictive importance and effect magnitude rather than statistical hypothesis testing.

## **Reviewers comment 6**

**While browsing through the results, a fundamental issue seems to be the disconnection from the behavioral theories that were introduced as the theoretical foundation. While the discussion appears to largely focus on four built environment characteristics. Due to this disconnection, the analysis and interpretations were largely simplified by overlooking the attitudinal dimensions and contradicts with the initial motivation and questions raised. This undermines the theoretical foundation and weakens the depth of analysis. I am not sure how this can be omitted in the first place.**

## **Response to comment 6**

Thank you very much for this insightful and constructive comment. We fully agree that a clear linkage between the behavioral theories and the empirical findings is essential for ensuring theoretical consistency and analytical depth. In the revised manuscript, we have carefully strengthened this connection throughout the results interpretation and conclusion sections.

Specifically, Section 4.2 (Significance of the threshold effect and its spatial distribution) has been revised to explicitly integrate the Theory of Planned Behavior (TPB) and the Technology Acceptance Model (TAM) into the interpretation of the nonlinear relationships. A leading paragraph has been added at the beginning of the section to clarify that objective built environment attributes influence commuting intention primarily through perception-based mechanisms, including perceived usefulness, perceived ease of use, attitudes, subjective norms, and perceived behavioral control. This statement establishes the theoretical bridge between environmental variables and behavioral responses.

Furthermore, for each key variable (population density, distance to the nearest bus stop, road network density, and distance from the city center), additional behavioral interpretations have been incorporated. The threshold effects are now explained not only from spatial or infrastructural perspectives but also from psychological mechanisms consistent with TPB and TAM constructs. For example:

Population density is interpreted in relation to subjective norms, perceived usefulness, and comfort perception.

Bus stop proximity is linked to perceived ease of use and perceived behavioral control through first- and last-mile accessibility.

Road network density is discussed in terms of comparative modal perception and perceived convenience.

Distance from the city center is interpreted through perceived effort, travel burden, and cognitive evaluation of time savings.

These revisions demonstrate that the observed nonlinear effects reflect behavioral responses mediated by psychological perceptions rather than purely physical relationships.

In addition, the conclusion and policy implication sections have been revised to reiterate that built environment characteristics influence commuting willingness through both spatial constraints and behavioral perception pathways, thereby reinforcing the theoretical coherence of the study.

It should also be noted that the primary objective of this research is to examine nonlinear effects and spatial heterogeneity of built environment factors on suburban railway commuting willingness using machine-learning-based modeling approaches. Therefore, TPB and TAM are adopted mainly as interpretive theoretical lenses rather than as full structural behavioral models. We have clarified this positioning in the revised manuscript to avoid potential misunderstanding. **The revised text are as follows (see Lines360–368，380-388，410-419，431-437，445-453，651-663，707-710 in manuscript)：**

At the outset of Section 4.2, it is explained that built environment factors influence residents' willingness to commute via the Loop Line Railway by affecting their perceptions, attitudes, and other related aspects:

It should be noted that, according to the Theory of Planned Behavior (TPB) and the Technology Acceptance Model (TAM), objective environmental attributes influence behavioral intention primarily by shaping individuals’ perceptions, attitudes, and perceived behavioral control[51]. In the context of suburban railway commuting, built environment conditions such as accessibility, connectivity, and spatial location may alter residents’ perceived usefulness, perceived ease of use, and perceived convenience of the Loop Line Railway, which subsequently affect their commuting willingness. Therefore, the non-linear relationships identified in this section can also be understood as behavioral responses mediated through psychological mechanism.

In the discussion of population density, we added the following theoretical interpretation to connect the threshold effect with TPB and TAM mechanisms:

From a behavioral perspective, population density also influences commuting willingness through psychological pathways. Higher density is often associated with better service frequency, stronger social visibility of public transport use, and more pronounced collective travel norms, which may enhance subjective norms and perceived usefulness of the Loop Line Railway[56]. In contrast, excessive density may reduce perceived comfort and negatively affect attitudes toward rail commuting[57]. Therefore, the observed threshold effect reflects not only spatial constraints but also changes in psychological perceptions and attitudes, consistent with TPB and TAM mechanisms.

In the discussion of the nearest bus stop, we added the following theoretical interpretation to connect the threshold effect with TPB and TAM mechanisms:

From the perspective of behavioral theory, improved feeder accessibility can enhance perceived ease of use and perceived behavioral control by reducing transfer difficulty and uncertainty[60]. When residents perceive multimodal connections to be convenient and reliable, their confidence in using the Loop Line Railway increases, thereby strengthening commuting intention. Conversely, longer distances to feeder transit may reduce perceived accessibility and increase psychological resistance to rail commuting. This mechanism further demonstrates that built environment factors influence commuting willingness through perception-based pathways, consistent with TPB constructs.

In response to the reviewer’s concern regarding the connection between empirical findings and behavioral theories, we have added a theoretical interpretation in the discussion of road network density to clarify how the observed non-linear relationship can be explained through TPB and TAM constructs:

Behaviorally, increased road connectivity may enhance perceived convenience and perceived behavioral control associated with reaching stations[63]. However, overly developed road systems may strengthen the perceived competitiveness of private cars or ride-hailing services, thereby weakening the perceived usefulness of rail commuting. This suggests that the observed non-linear relationship reflects changes in comparative modal perception rather than purely infrastructural effects.

In the discussion of the distance from the city center, we further incorporated a behavioral interpretation to explain the identified threshold effect through the psychological mechanisms proposed by TPB and TAM:

From a psychological perspective, spatial distance from the city center influences perceived usefulness and perceived effort associated with commuting. Residents located farther from employment centers may perceive higher travel burdens and uncertainty, which negatively affect attitudes toward rail commuting. However, in certain contexts, the rapid travel advantage of suburban rail may partially offset this spatial disadvantage, thereby enhancing perceived usefulness. These findings indicate that spatial location influences commuting willingness through cognitive evaluations of time savings and travel convenience.

Furthermore, in the conclusion section, we elaborate on the connection between the findings and the theoretical framework:

（4）Furthermore, the findings of this study also provide empirical support for the behavioral mechanisms proposed by the Theory of Planned Behavior (TPB) and the Technology Acceptance Model (TAM). The results indicate that built environment characteristics do not influence commuting willingness solely through physical accessibility constraints, but also indirectly by shaping residents’ perceptions of convenience, usefulness, and travel effort. Improvements in population density, feeder accessibility, and road connectivity may enhance perceived usefulness, perceived ease of use, and perceived behavioral control associated with the Loop Line Railway, thereby strengthening commuting intention. Conversely, unfavorable spatial conditions may reduce perceived advantages and increase psychological resistance to rail commuting. These findings highlight the importance of integrating objective spatial planning with perception-oriented service improvements to promote suburban railway utilization.

The role of theory is emphasised in the recommendations section: From a behavioral perspective, optimizing built environment conditions around stations not only improves objective accessibility but also enhances perceived usefulness and perceived behavioral control, thereby strengthening residents’ willingness to use suburban railways for commuting.

## **Reviewers comment 7**

**The figures are still in poor quality.**

## **Response to comment 7**

Thank you very much for the reviewer’s careful comments regarding the figure quality. We sincerely apologize for the inconvenience caused by the insufficient resolution in the previous version.

In this revision, all figures have been re-exported directly from the original software using high-resolution settings and have been uploaded as separate files in TIFF format to ensure optimal clarity during the production process. We have carefully checked all figures in the revised manuscript to confirm that labels, legends, and graphical elements are clearly readable.

Should the images remain unclear, we have uploaded all images to a public database. You may visit https://doi.org/10.6084/m9.figshare.31429004 to view the clear images. We hope that the updated figures now meet the journal’s publication standards.

***Responses to comments from Reviewer #* 2**

## **Reviewers comment 1**

**Although the authors have provided a link to the dataset, I was unable to access it during the review process. I kindly suggest that the authors verify whether the link is available and ensure that the data can be accessed by external users. Making the dataset reliably accessible would enhance the transparency of the study.**

## **Response to comment 1**

Thank you very much for the reviewer’s helpful suggestion regarding the data accessibility. We sincerely apologize for the inconvenience caused by the inaccessible link in the previous version.

We have now verified and updated the dataset link to ensure that it can be accessed by external users without any restrictions. The dataset has been made publicly available at the following repository:

https://doi.org/10.6084/m9.figshare.31146256

Access permissions have been set to allow open access, and the data can now be downloaded directly.
