# Supplementary material for: Spatial heterogeneity of the nonlinear impact of built environment on suburban railway commuting willingness: Taking Guiyang Loop Line Railway as an example
Source: PLoS One. 2026 Apr 7;21(4):e0346358. doi: 10.1371/journal.pone.0346358 (PMC13056205; doi:10.1371/journal.pone.0346358)
Supplement: Attachment — Submitted filename: Response_to_Reviewers_auresp_3.docx [file pone.0346358.s005.docx]

**Point-to-Point Responses to Reviewers**

The authors sincerely thank the Editor and the reviewers for their continued time and constructive comments on the revised manuscript. We have carefully addressed all comments and revised the manuscript accordingly. A detailed, point-by-point response is provided below, and the corresponding revisions have been indicated in the manuscript.

***Responses to comments from Reviewer #* 1**

## **Reviewers comment 1**

**When making important statements, e.g., defining suburban railway, the unsatisfactory ridership of suburban railway, etc., it is important to cite supporting literature or document and avoid overstatements. Please carefully check the manuscript throughout.**

## **Response to comment 1**

Thank you for this valuable comment. We carefully reviewed the entire manuscript and revised statements that lacked sufficient supporting references. Specifically, additional literature has been cited when defining suburban railways and when discussing the ridership performance of suburban railway systems. Furthermore, several expressions that might be interpreted as overstatements were moderated to ensure a more cautious and evidence-based presentation. These revisions help improve the academic rigor and reliability of the manuscript. **The revised text are as follows (see Lines 2-5、 170-172、178-181 in manuscript)**

Suburban railways, often termed commuter rail or suburban rail, are categorized as a form of passenger rail system positioned between urban rail transit (e.g., metro systems) and intercity railways; they are characterized by commuter-oriented services, relatively high operating speeds, and large transport capacity[1].

As of October 2024, the density of the urban road network was approximately 6.7 km/km², lower than the national recommended standard of 8 km/km²[43].

The Guiyang Loop Line Railway, China’s first urban loop suburban railway, was officially opened on March 30, 2022. The line has a total length of approximately 113 km with 17 stations, forming an important transportation corridor connecting multiple residential districts and urban functional areas and supporting the development of a “one-hour commuting circle” [44].

## **Reviewers comment 2**

**The forms of certain citations do not align with English writing, for example, line 138: “Jia Fang demonstrated that built…” and “Yafei Xi identified spatiotemporal non-linear relationships”—We usually only use surnames when citing previous research. There are plenty other examples with similar errors, which require careful correction.**

## **Response to comment 2**

Thank you for this helpful comment. We have carefully reviewed the manuscript and revised the citation format to conform to standard English academic writing conventions. Specifically, only the authors’ surnames are now used when referring to previous studies in the text. Similar issues throughout the manuscript have been systematically corrected, and the manuscript has been carefully proofread to ensure consistency in citation style. **The revised text are as follows (see Lines 138-139、144 in manuscript)**

For example, Jia demonstrated that built environment attributes such as block size and intersection density are strongly associated with residents’ mode choice decisions[38];

Similarly, Xi identified spatiotemporal non-linear relationships between metro station built environments and passenger flows, showing that factors such as population density and parking facility density exert significantly non-linear impacts on station-level ridership[40].

## **Reviewers comment 3**

**Sections 5 and 6 should be restructured. Particularly, Section 5 should discuss the key findings and actionable implications, while Section 6 should draw the conclusions and identify limitations for future works.**

## **Response to comment 3**

Thank you for this helpful suggestion. In accordance with the reviewer’s comment, Sections 5 and 6 have been restructured to improve the organization of the manuscript. Section 5 now discusses the key findings of the study and highlights their practical implications. Section 6 summarizes the main conclusions and identifies the limitations of the study as well as directions for future research. This restructuring enhances the logical flow of the paper. **The revised text are as follows (see Lines 594-712 in manuscript)**

5 Disscussion and Policy Implication

Drawing on the spatial analysis of threshold effects and non-linear impacts of built environment factors, this study proposes the following recommendations to address the low ridership and underutilisation of the Guiyang Loop Line Railway, and to provide practical insights for station planning and the development of suburban railways in other cities.

(1) The results indicate that the commuting function of the Loop Line Railway depends heavily on the presence of stable and sufficiently concentrated commuting willingness. In areas with high population density and strong job - housing concentration, the Loop Line Railway is more suitable as a frequent and reliable commuting mode, and its commuter-oriented function should be prioritised. In contrast, in peripheral areas characterised by lower population density and dispersed residential patterns, changes in population scale alone are unlikely to meaningfully enhance the commuting attractiveness of the Loop Line Railway. In such contexts, improving feeder connections and reducing overall commuting time represent more effective strategies for strengthening its relative advantages.

(2) For suburban and peripheral stations, priority should be given to improving the accessibility of bus feeder services rather than expanding the overall scale of bus supply. Rather than increasing the number of bus routes, more effective measures include optimising the spatial distribution of bus stops, shortening walking distances to the nearest bus stop, and providing dedicated feeder bus services to railway stations. These measures can reduce first- and last-mile transfer costs and, in turn, enhance the commuting attractiveness of the Loop Line Railway.

(3) The model results show that the non-linear effects of road network density on commuting willingness are mainly concentrated in suburban and peripheral areas. Accordingly, road network improvements should focus on areas where transport infrastructure is relatively underdeveloped. Measures such as improving local street networks, reducing detour distances, and enhancing road connectivity can increase station accessibility and facilitate residents’ access to railway stations, thereby encouraging the use of the Loop Line Railway for commuting. In contrast, in central urban areas where road networks are already highly developed, further increases in road density may yield limited benefits. Instead, measures such as dedicated access lanes for railway transfers and enhanced traffic flow management can be adopted to reduce congestion and improve transfer efficiency.

(4) The Loop Line Railway plays an important role in mitigating the disadvantages associated with long commuting distances in peripheral areas. For residents living far from the urban core, commuting distance and time costs constitute major constraints on railway use. In these areas, the cross-regional rapid accessibility provided by the Loop Line Railway is more likely to be strongly perceived and valued. Accordingly, in the process of optimising urban spatial structure, the Loop Line Railway can serve as an important instrument for improving commuting conditions in peripheral areas and promoting shifts in commuting modes. However, given the relatively low level of development in these areas, particular attention should be paid to strengthening feeder transport facilities and reducing overall commuting time. From a behavioral perspective, optimizing built environment conditions around stations not only improves objective accessibility but also enhances perceived usefulness and perceived behavioral control, thereby strengthening residents’ willingness to use suburban railways for commuting.

6 Conclusions and Limitations

6.1 Conclusions

Based on survey data on residents’ willingness to commute via the Guiyang Loop Line Railway, this study systematically examines the non-linear effects of built environment factors on commuter willingness and their spatial heterogeneity. The main conclusions are as follows:

(1) Population density, distance to the nearest bus stop, road network density, and distance from the city center represent the four built environment factors with the most substantial impact on commuting willingness. Specifically, population density and road network density exert a positive influence, whereas distance to the nearest bus stop and distance from the city center show an inverse correlation with commuting propensity for the Loop Line Railway.

(2) The non-linear dependence of commuting willingness on the built environment exhibits strong spatial non-stationarity along the Loop Line Railway. The significance of threshold effects and the intensity of non-linear impacts associated with different built environment factors vary substantially across space, forming a clear gradient from the urban core to suburban and peripheral areas. Compared with the central urban area, residents living around suburban and peripheral stations show greater sensitivity to changes in feeder bus conditions, road network connectivity, and commuting distance, which results in more pronounced threshold effects and stronger non-linear responses.

(3) The mechanisms through which built environment factors influence commuting willingness differ across spatial contexts, with peripheral areas showing greater potential for improving commuting willingness. Specifically, the threshold effects and non-linear impacts of population density are primarily concentrated around central urban stations, indicating that in areas with highly concentrated commuting willingness, changes in population scale have stronger marginal effects on willingness to commute via the Loop Line Railway. In contrast, the non-linear effects of distance to the nearest bus stop and road network density are more pronounced around suburban and peripheral stations, indicating that in areas with relatively underdeveloped public transport and road infrastructure, first- and last-mile connection conditions and station accessibility represent key constraints on using the Loop Line Railway for commuting. In addition, the non-linear effect of distance to the city center is most pronounced at stations located farther from the urban core, suggesting that the Loop Line Railway has greater potential to mitigate the spatial disadvantage associated with long commuting distances in peripheral areas.

（4）Furthermore, the findings of this study also provide empirical support for the behavioral mechanisms proposed by the Theory of Planned Behavior (TPB) and the Technology Acceptance Model (TAM). The results indicate that built environment characteristics do not influence commuting willingness solely through physical accessibility constraints, but also indirectly by shaping residents’ perceptions of convenience, usefulness, and travel effort. Improvements in population density, feeder accessibility, and road connectivity may enhance perceived usefulness, perceived ease of use, and perceived behavioral control associated with the Loop Line Railway, thereby strengthening commuting intention. Conversely, unfavorable spatial conditions may reduce perceived advantages and increase psychological resistance to rail commuting. These findings highlight the importance of integrating objective spatial planning with perception-oriented service improvements to promote suburban railway utilization.

6.2 Limitations and Future Research Directions

Although this study provides a systematic analysis of the non-linear effects and spatial heterogeneity of built environment factors on commuting willingness, several limitations remain that warrant further investigation.

First, this research is limited to the Guiyang Loop Line Railway as a single case studay. As a typical mountainous city, Guiyang possesses distinctive topographical features, urban spatial structures, and a unique rail transit development stage. Therefore, caution is required when generalizing the findings to cities with divergent geographical conditions or development trajectories. Future studies should incorporate comparative analyses across multiple cities or railway lines to validate the robustness and generalizability of the conclusions.

Second, the analysis relies on cross-sectional survey data, which captures residents’ stated commuting willingness at a single temporal point. Consequently, the study cannot fully capture dynamic shifts in commuting willingness resulting from built environment improvements or operational adjustments. Future research could benefit from integrating longitudinal survey data with objective travel data (e.g., smart card or mobile phone signaling data) to better examine the long-term behavioral impacts of built environment interventions.

Finally, data constraints limited the built environment measurement to indicators such as population density, road network density, and bus stop proximity. Other potentially relevant factors, such as pedestrian environment quality and public transport reliability, were not explicitly considered. Future studies should incorporate finer-grained spatial and environmental data to elucidate the mechanisms through which the built environment influences suburban railway commuting behavior.

***Responses to comments from Editor***

## **Reviewers comment 1**

**If the reviewer comments include a recommendation to cite specific previously published works, please review and evaluate these publications to determine whether they are relevant and should be cited. There is no requirement to cite these works unless the editor has indicated otherwise.**

**Please review your reference list to ensure that it is complete and correct. If you have cited papers that have been retracted, please include the rationale for doing so in the manuscript text, or remove these references and replace them with relevant current references. Any changes to the reference list should be mentioned in the rebuttal letter that accompanies your revised manuscript. If you need to cite a retracted article, indicate the article’s retracted status in the References list and also include a citation and full reference for the retraction notice.**

## **Response to comment 1**

Thank you for this reminder. We have carefully reviewed the reference list to ensure that all references are complete, accurate, and relevant to the study. A small number of additional references have been incorporated where appropriate to strengthen the literature support of the manuscript. We also confirmed that none of the cited references have been retracted.
